# Eu Doping in the GdCd_7.88_ Quasicrystal and Its Approximant Crystal GdCd_6_

**DOI:** 10.1021/acs.inorgchem.3c04500

**Published:** 2024-03-01

**Authors:** Fernand Denoel, Yu-Chin Huang, Neha Kondedan, Andreas Rydh, Cesar Pay Gómez, Roland Mathieu

**Affiliations:** † Department of Materials Science and Engineering, Uppsala University, Box 35, Uppsala, 751 03, Sweden; ‡ Department of Chemistry-Ångström Laboratory, Uppsala University, Uppsala 751 21, Sweden; § Department of Physics, Stockholm University, Stockholm 10691, Sweden

## Abstract

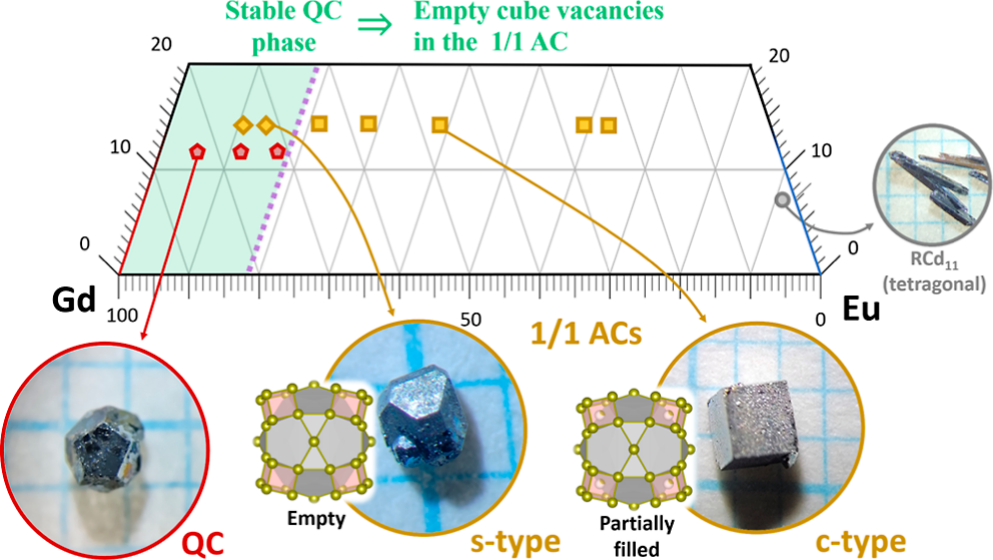

The effect of Eu doping in the Tsai quasicrystal (QC) GdCd_7.88_ and its periodic 1/1 approximant crystal (AC) GdCd_6_ are investigated. This represents the first synthesis of Eu-containing stable QC samples, where three samples with the final composition Gd_1–*x*_Eu_*x*_Cd_7.6±α_ at Eu doping concentrations *x* = 0.06, 0.13, and 0.19 are obtained (α ∼ 0.2). They are compared to two 1/1 ACs with compositions Gd_1–*x*_Eu_*x*_Cd_6_ (*x* = 0.12, 0.16). In addition, a new type of 1/1 AC, differing only by the inclusion of extra Cd sites unique to the Eu_4_Cd_25_ 1/1 AC, has been discovered and synthesized for the concentrations Gd_1–*x*_Eu_*x*_Cd_6+δ_ (*x* = 0.25, 0.33, 0.45, 0.69, 0.73, and 0 < δ ≤ 0.085). Due to the preferred cube morphology of its single grains, we refer to them as c-type 1/1 ACs and to the conventional standard ones as s-type. In both QCs and s-type ACs, the Eu content appears to saturate at a concentration of ∼20%. On the other hand, any Gd| Eu ratio is allowed in the c-type ACs, varying continuously between GdCd_6_ and Eu_4_Cd_25_. We describe and contrast the changes in composition, atomic structure, specific heat, and magnetic properties induced by Eu doping in the quasicrystalline phase and the s-type and c-type 1/1 ACs. By comparing our results to the literature data, we propose that the occupancy of the extra Cd sites can be used to predict the stability of Tsai-type quasicrystalline phases.

## Abstract 2

Icosahedral quasicrystals (i-QCs) constitute an intriguing state of matter with an aperiodic structure in all three dimensions. Approximant crystals (ACs) possess the same local atomic environment but are periodic crystals. All stable Tsai-type i-QCs possess AC counterparts, but the reverse is not true. Through a careful structural analysis of Eu-doped Gd–Cd i-QCs and ACs, we establish a link between AC structure and the existence of stable QCs, which can be generalized to other systems.

## Introduction

Quasicrystals (QCs) constitute an unconventional state of matter that has been thoroughly researched since their discovery 40 years ago.^1−3^ Tsai-type QCs are particularly well suited for understanding the interplay between magnetism and quasiperiodic lattices since they include lanthanide elements with strong spin moments associated with 4f magnetism and surrounded by nonmagnetic elements, such as Cd,^4^ or a mixture of monovalent and trivalent atoms. Stable examples of QCs can be obtained using the (Ag,In) pair,^5^ but metastable samples obtained from fast quenching have also been reported, using (Au,Al)^6^ or (Au,Ga)^7^ as the nonmagnetic elements. Long-range magnetic order has been evidenced for the first time in the latter with Gd and Tb as the rare earth magnetic elements. Approximant crystals (ACs) are periodic counterparts made of the same building block (Tsai cluster) which are arranged in a body center cubic (bcc) structure in the case of 1/1 ACs, the most common variety. One of the main obstacles to a complete understanding of magnetic behavior in quasiperiodic lattices is the presence of chemical disorder in all known QCs including a nonzero 4f magnetic moment. In general, the Tsai cluster in QCs only possesses lanthanide sites mixed with nonmagnetic elements and can be seen as a diluted system compared to the perfect quasiperiodic lattice, with a formula unit close to MCd_7.5_ (M = Gd–Tm, Y) compared to MCd_5.7_ in Tsai-type QC systems exhibiting a perfect chemical order for the divalent M = Yb, Ca.^8^ However, since Yb^2+^ is nonmagnetic under ambient pressure,^9^ it is not possible to study the impact of magnetic elements without chemical disorder-induced dilution on a quasiperiodic lattice. By doping the Gd–Cd QC with Eu ions, we can infer what the exact conditions for a perfect chemical order are and observe if a compromise can be achieved to increase the occupancy of magnetic elements.

Most lanthanide elements are found to be stable in the trivalent state R^3+^, as observed in all Cd-based QCs except with Yb. Europium is the only other lanthanide that has been shown to exhibit a divalent Eu^2+^ state in its 1/1 ACs, but QC formation appears to be impossible for all the R lanthanides with an atomic number below that of Gd (i.e., R = La–Eu), with one possible reason being their larger size.^12^ The Hume–Rothery criterion for stability gives a satisfying description of the chemical composition of QCs based on the valence of its constituent atoms.^13^ The average valence electron value per atom e/a has been empirically found to be constrained to values close to 2 in Tsai-type QCs. With the exception of Yb and Ca, all binary Tsai QCs possess a trivalent atom on the rare-earth site, which must increase the e/a ratio. From this perspective, it is assumed that the chemical disorder with divalent Cd^2+^ on the icosahedral shell is a compromise quasicrystalline systems accept to stay within the stability region where they can form. In the case of binary Cd-based QCs, the upper limit is e/a ∼2.12,^5^ but it can be slightly expanded toward e/a ∼2.15 in the R–Cd–Mg ternary QC systems.^14^ A binary Eu–Cd QC may be unachievable, but by taking the existing binary GdCd_7.88_ as a starting point, one can *a priori* increase the Eu content up to a certain limit. On the other hand, in the 1/1 AC case, both the GdCd_6_ and Eu_4_Cd_25_ 1/1 ACs exist, with the difference of composition being explained by the larger Eu^2+^ ions enlarging the unit cell enough to allow extra Cd sites to populate interstitial sites in an ordered manner (see the Results Section for more details about the sites). For that reason, the Eu_4_Cd_25_ 1/1 AC is a superstructure of the conventional 1/1 AC, with a doubling of the cubic unit cell in all directions.^15^ Previous studies of doping in the GdCd_6_ 1/1 AC include the dilution by nonmagnetic Y atoms at the Gd sites, where the Néel temperature was found to be suppressed for small doping.^16^

In the following, we describe the synthesis process of the ternary Gd–Eu–Cd QC and AC phases obtained from a mixture of Gd and Eu as the lanthanide ions and, more specifically, introduce the two distinct 1/1 AC phases obtained: the standard (s-type) 1/1 ACs with conventional morphology and the cube-shaped (c-type) 1/1 ACs. We compare the structural, magnetic, and specific heat data from Eu-doped samples and undoped binary samples in each case and discuss how they are impacted by the Eu-doping.

## Experimental Methods

Samples were synthesized by using the self-flux method. For the starting compositions, we used Chempur granules of Eu, Gd, and Cd with high purities of 99.9, 99.999, and 99.999%, respectively. The starting nominal Eu concentrations were (Gd_1–*y*_Eu_*y*_)_0.8_Cd_99.2_, with *y* = 0.3, 0.4, and 0.5 for the QC. In addition, one Eu-doped Ho–Cd QC was synthesized with the starting composition (Ho_0.6_Eu_0.4_)_0.8_Cd_99.2_. All QCs were centrifuged at a temperature range from 335 to 360 °C. In addition, the synthesis of seven 1/1 ACs was performed from starting nominal compositions in the range (Gd_1–*y*_Eu_*y*_)_2–10_Cd_98–90_ for *y* = 0.3–0.7, obtained at a centrifugation temperature range from 390 to 554 °C. Note that since the starting material composition and final composition differ significantly in most of the synthesized samples, we use the parameter *y* to refer to the former and *x* to refer to the latter throughout the text. The starting materials were placed in an alumina crucible and sealed in stainless steel ampules under an inert Ar atmosphere. The ampules were first heated to 700 °C for 10 h and maintained for another 5 h in order to obtain homogeneous melts. The temperature was then slowly lowered at a rate of 2 °C/h from about 650 °C to their final temperatures, ranging from 460 to 550 °C, for the 1/1 ACs and at a rate of 1 °C/h from 600 °C to the final temperatures, ranging from 355 to 380 °C, for the QCs. The samples were kept for annealing at their final temperature for 24–48 h before the remaining flux was centrifuged to extract the single-crystal grains. A complete summary of the syntheses can be found in the Supporting Information, Table S1, with CIF files available.

Sample characterization was performed using powder X-ray diffraction (PXRD) with Cu Kα radiation (λα1 = 1.540598 Å and λα2 = 1.544390 Å). Room (293 K)- and low (100 K)-temperature single-crystal X-ray diffraction (SCXRD) measurements were performed using a Bruker D8 single-crystal X-ray diffractometer using an Incoatec Microfocus Mo X-ray Source with wavelength Kα = 0.71073 Å and an APEX II CCD area detector at room temperature. In order to collect high-quality SCXRD data, we cut well-faceted grains into ∼50–100 μm pieces to minimize absorption effects. All data sets were collected using 40 s of exposure time and a step size of 0.3°. Data reduction was performed in APEX III software.^17^ All the structural refinements were performed in Jana 2006 software,^18^ and the crystal structure was visualized using the software VESTA.^19^

The unit cell parameter of the AC samples was indexed from the PXRD data in CheckCell software^20^ and compared to SCXRD results. The sample composition was asserted using two techniques, scanning electron microscopy (SEM) equipped with energy-dispersive X-ray spectroscopy (EDX) and the inductively coupled plasma optical emission spectroscopy (ICP-OES) method, in order to distinguish the chemically similar Gd and Eu elements. The ICP-OES analysis was performed by MikroLab Mikroanalytisches Laboratorium Kolbe. The EDX acquisition was performed on polished samples and collected with an acceleration voltage of 20 kV with a working distance of 8 mm. We performed differential scanning calorimetry (DSC) on one of the QC samples and each of the s-type and c-type 1/1 ACs in a NETZSCH STA 449 F3 Jupiter at a temperature of up to 850 °C with a heating/cooling rate of 10 °C/min to determine the formation temperature of the sample. The DSC measurements were performed under an argon flow of 40 mL per min. The specific heat measurements were acquired by using a Bluefors dilution refrigerator equipped with a superconducting magnet with a maximum field of 12 T. A differential membrane-based nanocalorimeter with an operating range down to 100–200 mK was used for the data collection, with samples of typical dimensions 100 × 100 × 50 μm^3^. All of the dc magnetization data were acquired on single crystals of the various phases using an MPMS XL SQUID from Quantum Design, Inc.

## Results

**Figure 1:**
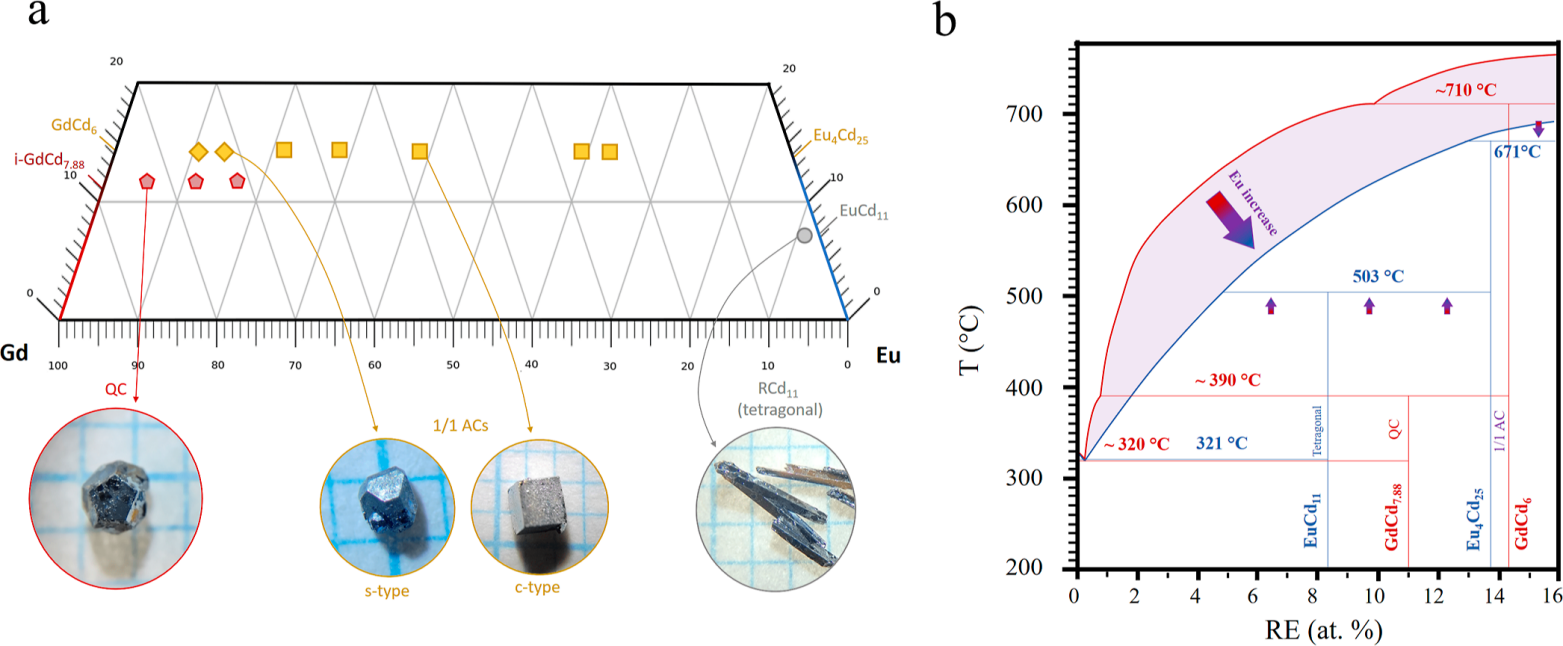
(a) Truncated triangular phase diagram. The three relevant crystal phases from the Cd-rich part of the triangular phase diagram are the QC (red), s- and c-type 1/1 ACs (yellow), and tetragonal (gray). They are represented by pentagons, diamonds, squares, and circles, respectively. (b) Superposition of the pure binary Gd–Cd (red) and Eu–Cd (blue) phase diagrams; the arrows indicate the qualitative changes of the phase diagram as the Eu concentration increases. The phase diagrams in (b) were taken from refs (10 and 11).

During the initial synthesis attempts of Eu-doped QCs, we observed the formation of an unwanted secondary tetragonal phase with a crystal structure-type BaCl_11_ (space group: *I*4_1_/*amd*), coexisting with the QC phase (see Figure S1). Raising the final temperature, however, permitted us to synthesize single-phase QCs, i.e., without a secondary tetragonal phase. As the nominal starting composition (Gd_1–*y*_Eu_*y*_)_0.8_Cd_99.2_ is shifted to the Eu-rich part of the phase diagram, the formation of the QC phase is rendered impossible at a Eu concentration above *y* = 0.6, but macroscopic single grains become difficult to achieve above *y* = 0.5 since the liquidus line is lowered as the Eu concentration increases, making the temperature range where QCs form narrower (see Figure 1b).

We also synthesized a total of seven Eu-doped 1/1 ACs. Two of the samples were obtained with a standard morphology and are referred to as s-type approximant crystal (s-AC) in the text. All other 1/1 ACs synthesized showed a cube-shaped morphology. We refer to them as c-type approximant crystals (c-ACs). The main difference in synthesis between the two types is the final temperature, where a low final temperature of 390–460 °C selects the s-AC, whereas a higher range, 505–554 °C, selects the c-AC type. The typical facets of the aforementioned phases and where they are located in the Gd–Eu–Cd triangular phase diagram can be found in Figure 1a.

**Figure 2:**
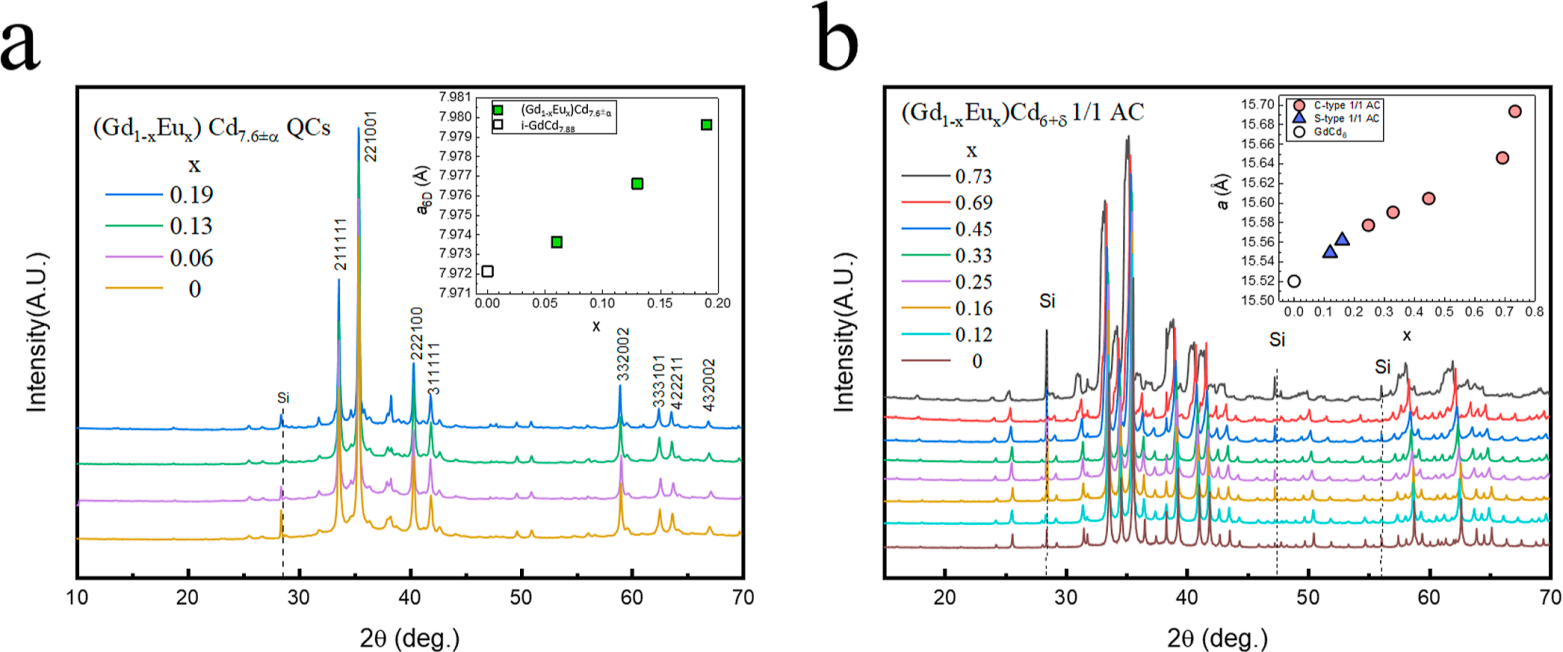
Powder XRD patterns of Eu-doped samples for (a) the QCs and (b) the s- and c-type 1/1 ACs. The insets show the hyperspace *a*_6D_ parameters of the QCs and the lattice parameters of the 1/1 ACs, respectively.

In terms of compositions, the ICP-OES and EDX analyses (see Tables S2 and S3) reveal a saturation of the Eu concentration, which does not exceed *x* = 0.19 in the case of QCs regardless of the starting material Eu content, yielding QCs with *x* = 0.06, 0.13, and 0.19 and a Cd content equal to 7.62, 7.4, and 7.54, respectively. We denote the doped QCs as Gd_1–*x*_Eu_*x*_Cd_7.6±α_, with α ∼ 0.2. A similar saturation behavior at *x* = 0.16 is observed for the s-type 1/1 ACs (*x* = 0.12 and 0.16). In both cases, the Gd/Eu ratio of the starting materials was *y* = 0.5. However, the c-type 1/1 ACs present a larger Eu content: *x* = 0.25, 0.33, 0.45, 0.69, and 0.73. The Cd content is determined from the SCXRD data (see Table 1), yielding the Gd_1–*x*_Eu_*x*_Cd_6+δ_ composition. No sign of saturation upon Eu-doping appeared in the case of c-type 1/1 ACs, with rare-earth final concentrations found to be closer to the starting material concentration (see Figure S2). The gap between the starting material and final composition is explained by the remaining Eu being left in the centrifuged flux after synthesis instead of being incorporated in the crystals grown. Regarding the tetragonal BaCl_11_-type crystals obtained from the starting composition *y* = 0.5 and analyzed from ICP, we observed an opposite trend, with the saturating element being Gd in this case, at a relatively low concentration of 2%; i.e., the rare-earth concentration in tetragonal-phase samples only differs from that of the binary EuCd_11_ by a few percent regardless of the starting material composition. The structure characterization of the QCs was performed using PXRD,^21^ while for s- and c-type ACs, both PXRD and SCXRD were used. For all samples, we observed a systematic peak shift toward the low 2θ angle in the PXRD patterns obtained as the Eu concentration increases, indicating that the lattice/hyperlattice parameter is expanded (see Figure 2). We observe, however, a smaller peak shift, i.e., Tsai cluster expansion, in the QC samples compared to that in the 1/1 ACs upon doping. One can relate the 6D hyperlattice constant, *a*_6D_, to the 3D “real space” *a*_1/1_ lattice constants the 1/1 AC with the same Tsai cluster diameter would have by using the equation , where  is the golden ratio.^22^ The *a*_1/1_ equivalents from Eu-doped QCs will be compared to the lattice parameters of the Eu-doped 1/1 ACs later on. The SCXRD patterns of all five c-ACs and one of the s-ACs (*x* = 0.16) were also acquired for structure refinement.

**Table 1:** SCXRD Refined Parameters of the 1/1 ACs

refined comp.	Cd_6_ Eu_0.16_ Gd_0.84_	Cd_6.004_ Eu_0.25_ Gd_0.75_	Cd_6.012_ Eu_0.33_ Gd_0.67_^a^	Cd_6.027_ Eu_0.45_ Gd_0.55_	Cd_6.033_ Eu_0.69_ Gd_0.31_	Cd_6.085_ Eu_0.73_ Gd_0.27_
1/1 AC type	s-type	c-type	c-type	c-type	c-type	c-type
Eu conc. (*x*)	0.16	0.25	0.33	0.45	0.69	0.73
molar mass (g/mol)	830.9	830.8	831.3	832.4	831.8	837.4
temp. of meas. (K)	293	293	293	293	293	293
space group	*Im*3̅	*Im*3̅	*Im*3̅	*Im*3̅	*Im*3̅	*Im*3̅
*a* axis (Å) (SCXRD)	15.5672(6)	15.5792(11)	15.5845(3)	15.6033(5)	15.634(3)	15.6873(10)
*a* axis (Å) (PXRD)	15.5615(13)	15.5772(13)	15.590(4)	15.6056(50)	15.64598(215)	15.6890(52)
cell volume (Å^3^)	3772.5(3)	3781.3(5)	3785.11(13)	3798.8(2)	3821.0(11)	3860.5(4)
*Z*	24	24	24	24	24	24
calc. density (g/cm^3^)	8.7773	8.7567	8.7528	8.7322	8.675	8.6447
abs. coeff. (mm^–1^)	30.051	29.943	29.893	29.766	29.447	29.32
indep. reflections	1153	1069	1665	1068	1074	1086
obs. reflections	22,681	21,589	71,325	20,223	17,283	21,393
*R*_int_ (obs/all)	1.63/1.63	5.92/5.92	5.29/5.48	3.84/3.84	2.93/2.93	2.58/2.58
refined parameters	94	122	112	127	127	127
redundancy	19.671	20.196	42.838	18.935	16.092	19.699
R1 (obs/all)	0.186/0.190	0.0245/0.0245	0.022/0.0376	0.0231/0.0231	0.0197/0.0202	0.0198/0.0199
wR2 (obs/all)	0.599/0.599	0.0932/0.0932	0.0521/0.0548	0.0889/0.0889	0.0624/0.0634	0.0706/0.0721
GOF on *F*^2^ (obs/all)	2.43/2.45	3.93/3.93	1.62/1.75	3.53/3.53	2.48/2.50	2.98/3.04
Δρ_max_^′^·Δρ_min_^′^ (e/Å^3^)	1.03/–1.98	1.57/–1.29	4.74/–3.93	2.82/–1.59	2.00/–0.97	1.31/–1.63

a Acquisition in a different D8 venture single-crystal diffractometer with a Shutterless PHOTON III detector.

The s-AC sample was found to be isostructural to the GdCd_6_ 1/1 AC, whereas the c-ACs could not be satisfyingly described using only the atomic Wyckoff sites of the undoped sample. The single-crystal refinement results are listed in Table 1, and the refined positions are listed in Table 2. The atomic structure and composition differ between the two types of 1/1 ACs, with c-ACs having a slightly more Cd-rich composition, RCd_6+δ_, compared to their s-AC counterparts, as opposed to the conventional RCd_6_ formula unit per rare-earth atom. We note that the cadmium value per rare-earth atom of the c-ACs falls in between the limit values given by the undoped binary 1/1 AC samples GdCd_6_ and Eu_4_Cd_25_, i.e., 6 < 6 + δ < 6.25. The change in composition is explained by partially occupied extra Cd sites, unique to the Eu_4_Cd_25_ 1/1 AC. However, contrary to the pure Eu 1/1 AC, no superlattice reflections have been observed from SCXRD in either s- or c-type 1/1 ACs at room temperature, implying that the extra Cd sites at Wyckoff position 8c are not occurring in a periodic manner. Also, the low-temperature SCXRD of the c-AC with *x* = 0.45 acquired at 100 K did not show additional peaks either (see Figure S6). This implies the structural transition from cubic to monoclinic, space group *C*2/*c*, induced by an ordering of the Tsai clusters’ center tetrahedra occurring at *T*_s_ = 156 K in the undoped GdCd_6_,^4^ is suppressed by the Eu-doping, whereas the transition remained in Y-doped 1/1 AC samples at similar concentrations.^16^ The large ionic size difference between Eu^2+^ and Gd^3+^ can explain the suppression of the monoclinic transition.^23^

**Table 2:** SCXRD Refined Positions of the 1/1 ACs

atom	Wyck.	sample Eu conc. (*x*)	S.O.F.	*x*/*a*	*y*/*b*	*z*/*c*	U_equiv_
Gd/Eu	24g	*x* = 0.16 (s-type)	0.84/0.16	0.29962(2)	0.18864(2)	0	0.01860(8)
		*x* = 0.25	0.75/0.25	0.29965(3)	0.18858(3)	0	0.0156(1)
		*x* = 0.33	0.67/0.33	0.299585(16)	0.188458(15)	0	0.01267(7)
		*x* = 0.45	0.55/0.45	0.29955(2)	0.18839(3)	0	0.0147(1)
		*x* = 0.69	0.31/0.69	0.29948(2)	0.18839(2)	0	0.0190(1)
		*x* = 0.73	0.27/0.73	0.29914(2)	0.18836(2)	0	0.0194(1)
Cd1	24g	*x* = 0.16 (s-type)	0.33	0.0855(10)	–0.0639	0	0.205(8)
		*x* = 0.25	0.33	0.0838(8)	0.0701(8)	0	0.130(9)
		*x* = 0.33	0.33	0.0790(7)	0.0757(7)	0	0.111(3)
		*x* = 0.45	0.33	0.089(2)	0.056(3)	0	0.213(6)
		*x* = 0.69	0.33	0.0837(9)	0.0695(12)	0	0.136(5)
		*x* = 0.73	0.33	0.0825(9)	0.0686(10)	0	0.140(4)
Cd2	16f	*x* = 0.16 (s-type)	1	0.16143(6)	0.16143(6)	–0.16143	0.0324(2)
		*x* = 0.25	1	0.16140(9)	0.16140(9)	0.16140(9)	0.0300(4)
		*x* = 0.33	1	0.16129(5)	0.16129(5)	0.16129(5)	0.0286(2)
		*x* = 0.45	1	0.16102(10)	0.16102(10)	0.16102(10)	0.0357(5)
		*x* = 0.69	1	0.16038(8)	0.16038(8)	0.16038(8)	0.0450(4)
		*x* = 0.73	1	0.15768(12)	0.15768(12)	0.15768(12)	0.0619(4)
Cd3	24g	*x* = 0.16 (s-type)	1	0.09237(3)	0.24030(5)	0	0.04049(17)
		*x* = 0.25	1	0.09253(10)	0.24077(14)	0	0.0369(7)
		*x* = 0.33	1	0.09231(5)	0.24061(4)	0	0.0322(3)
		*x* = 0.45	1	0.09259(10)	0.24067(13)	0	0.0352(7)
		*x* = 0.69	1	0.09237(8)	0.24074(10)	0	0.0394(5)
		*x* = 0.73	1	0.09213(7)	0.24115(10)	0	0.0365(5)
Cd4	12d	*x* = 0.16 (s-type)	1	0	0.40670(5)	0	0.0388(2)
		*x* = 0.25	1	0	0.40693(17)	0	0.0357(9)
		*x* = 0.33	1	0	0.40694(4)	0	0.0325(2)
		*x* = 0.45	1	0	0.40718(16)	0	0.0340(9)
		*x* = 0.69	1	0	0.40736(11)	0	0.0378(7)
		*x* = 0.73	1	0	0.40763(11)	0	0.0355(7)
Cd5	48h	*x* = 0.16 (s-type)	1	0.20049(5)	0.34062(5)	0.11748(5)	0.0261(3)
		*x* = 0.25	1	0.20054(8)	0.34078(7)	0.11739(8)	0.0243(4)
		*x* = 0.33	1	0.20052(4)	0.34096(4)	0.11719(4)	0.0199(2)
		*x* = 0.45	1	0.20040(7)	0.34118(7)	0.11671(8)	0.0238(4)
		*x* = 0.69	1	0.20010(5)	0.34168(5)	0.11616(6)	0.0290(3)
		*x* = 0.73	1	0.19958(5)	0.34284(6)	0.11459(6)	0.0296(3)
Cd6	8c	*x* = 0.16 (s-type)					
		*x* = 0.25	0.013(6)	0.25	0.25	0.25	0.029(17)
		*x* = 0.33	0.036(3)	0.25	0.25	0.25	0.040(8)
		*x* = 0.45	0.080(8)	0.25	0.25	0.25	0.066(13)
		*x* = 0.69	0.098(5)	0.25	0.25	0.25	0.042(5)
		*x* = 0.73	0.256(6)	0.25	0.25	0.25	0.115(8)
Cd7	24g	*x* = 0.16 (s-type)	1	0.34550(3)	0.40385(3)	0	0.02162(11)
		*x* = 0.25	1	0.34551(4)	0.40377(4)	0	0.01894(18)
		*x* = 0.33	1	0.34549(2)	0.40377(2)	0	0.01593(10)
		*x* = 0.45	1	0.34549(4)	0.40371(4)	0	0.01810(17)
		*x* = 0.69	1	0.34559(3)	0.40382(3)	0	0.02268(13)
		*x* = 0.73	1	0.34604(3)	0.40405(3)	0	0.02270(13)
Cd8	12e	*x* = 0.16 (s-type)	1	0.5	0.30994(4)	0	0.02422(16)
		*x* = 0.25	1	0.5	0.30985(6)	0	0.0216(2)
		*x* = 0.33	1	0.5	0.30993(4)	0	0.01883(14)
		*x* = 0.45	1	0.5	0.30990(6)	0	0.0211(2)
		*x* = 0.69	1	0.5	0.30981(4)	0	0.02567(19)
		*x* = 0.73	1	0.5	0.30936(4)	0	0.0258(2)

**Figure 3:**
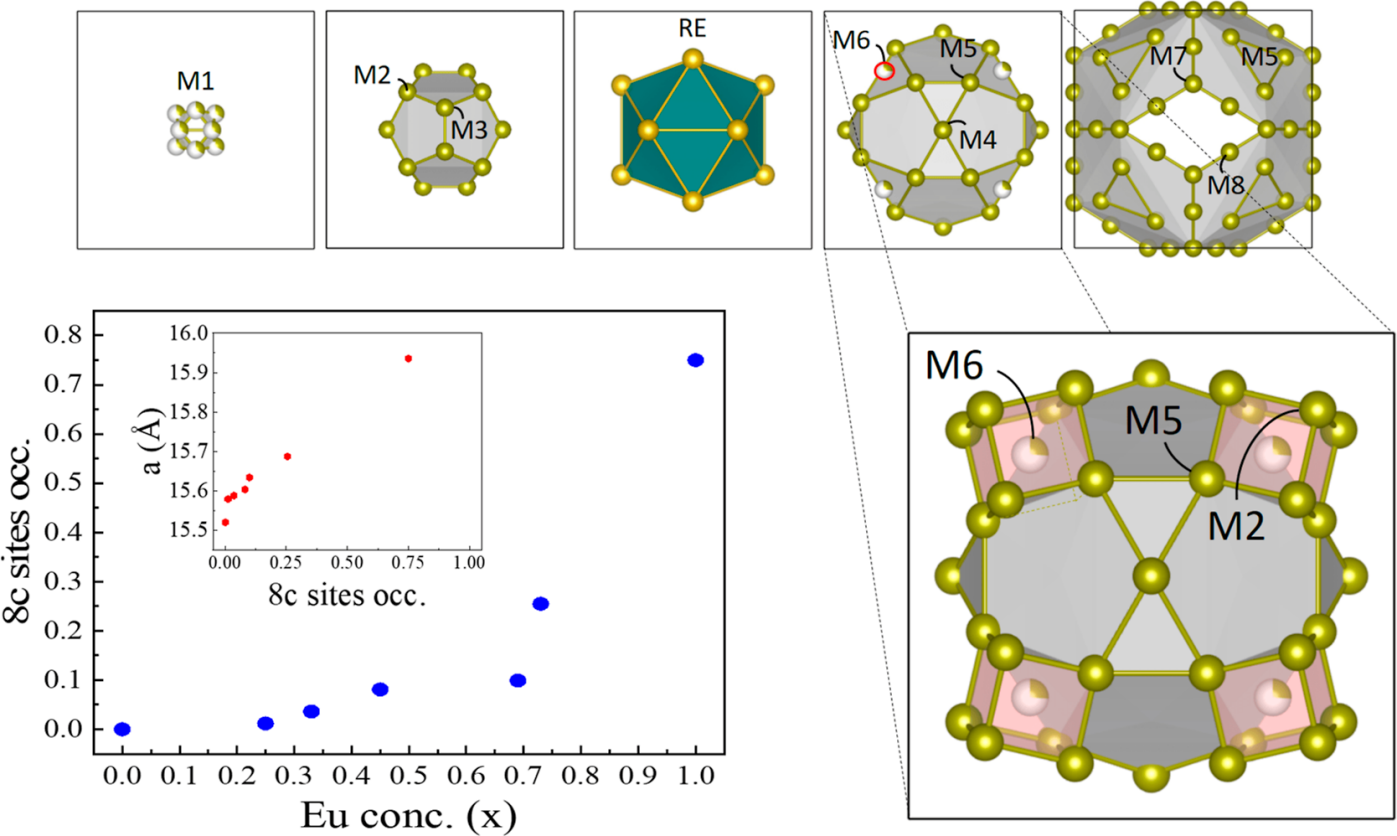
(Top) Concentric shells composing the Tsai cluster and corresponding nonequivalent atomic sites. The Wyckoff 8c site (M6 in the figure) is absent in the undoped GdCd_6_ and s-type 1/1 ACs but present in all c-type Eu-doped 1/1 ACs. From left to right: cuboctahedron (disordered tetrahedra), dodecahedron, rare-earth icosahedron, icosidodecahedron with partially occupied 8c site positions, and defect rhombic triacontahedron. (Bottom left) Occupancy of the 8c site as a function of the Eu concentration in the c-type 1/1 AC samples. The inset shows the corresponding dependence of the lattice parameter as a function of 8c site occupancy.

The GdCd_6_ 1/1 AC is isostructural to YCd_6_ and best described as a bcc packing of Tsai clusters in the unit cell with space group *Im*3̅ for both compounds. Mixing multiple rare earth magnetic elements within a 1/1 AC has only been investigated in the context of substitution with the nonmagnetic Y element, disrupting the original magnetic ordering.^16^ Owing to the different atomic size and chemical behavior of Cd compared to those of rare earth magnetic elements compatible with RCd_6_ 1/1 AC formation, rare-earth doping is only expected to occur on the icosahedron shell, leaving the Cd-based shells identical to the undoped structure. However, due to the large size of Eu^2+^ ions, extra Cd sites usually absent in the Cd-based 1/1 ACs appear in our Eu-doped c-type samples. The Eu_4_Cd_25_ 1/1 AC belongs to a different space group, owing to the alternating ordered–disordered inner tetrahedra from the Tsai cluster centers, as well as extra partially occupied Cd sites located between the icosidodecahedron and rhombic triacontahedron shells of the Tsai cluster along the (1 1 1) axes shown in Figure 3 for the c-type 1/1 AC unit cell structure. The Eu_4_Cd_25_ superstructure unit cell is displayed in Figure S5.

These structural differences make Eu_4_Cd_25_ a superstructure of the conventional 1/1 ACs, with space group *Fd*3̅ and a 2 × 2 × 2 supercell. The corresponding Wyckoff sites of these partially occupied interstitial Cd atoms are 8c when seen from the perspective of the conventional space group *Im*3̅ of most 1/1 ACs^24^ but correspond to the positions 16c and 32e when taken in the space group *Fd*3̅, unique to Eu_4_Cd_25_.^15^ Both Gd and Eu sit at the same atomic site in the icosahedral shell of the Tsai-type cluster. None of the structure solution methods from SCXRD can accurately resolve the correct Gd/Eu ratio from the SCXRD pattern alone since their atomic numbers are large and next to each other, with *Z* = 63 for Eu and *Z* = 64 for Gd. The different possible valence states of Eu further add complexity to the interpretation. Hence, in order to perform reliable crystal structure refinements and analyze the variation of the surrounding Cd element, we constrained the Eu/Gd ratio with analytical results from EDX (available in Table S1).

From a structural perspective, the binary GdCd_6_ and Eu_4_Cd_25_ 1/1 AC systems are different, and the former system has been reported to have a low-temperature structure phase transition to a monoclinic phase with space group *C*2/*c* at ∼156 K,^4,25^ associated with an ordering of the tetrahedra located at the Tsai cluster center. On the other hand, the Eu_4_Cd_25_ 1/1 AC already possesses ordered tetrahedra orientations, along with an ordered presence/vacancies of the interstitial sites 16c and 32e (defining the superstructure cell) compared to conventional 1/1 ACs. The atomic decoration in the Tsai-type cluster is shown in Figure 3, and the polyhedral arrangement and related atom position from the inner shell to the outer shell are as follows: the M1 site (Wyckoff 24g) located on the disordered tetrahedra, the M2 (Wyckoff 16f) and M3 (Wyckoff 24g) sites on the dodecahedron, M4 (Wyckoff 12d) being the rare-earth site on the icosahedron, the M5 site (Wyckoff 48h) on the icosidodecahedron, the M6 site (Wyckoff 8c) located between the icosidodecahedron and the rhombic triacontahedron, and, finally, the M5, M7 (Wyckoff 24g), and M8 (Wyckoff 12e) located on the outermost rhombic triacontahedron. Except for the M6 site unique to the c-AC, which is partially occupied, all the other Cd positions are fully occupied. Accounting for the M6 site (Wyckoff site 8c) at (0.25, 0.25, 0.25) gives the refined composition RCd_6+δ_ with δ = 0.004, 0.012, 0.027, 0.033, and 0.085, respective to the Eu concentrations *x* = 0.25, 0.33, 0.45, 0.69, and 0.73 (see Table 1), in which the s-AC at Eu saturation was given for comparison. The occupancy of the M6 interstitial site increases nonlinearly with the total Eu concentration (see Figure 3), while no occupancy was observed in the s-type 1/1 AC. The electron density isosurface surrounding the M6 site is given in Figure S4.

**Figure 4:**
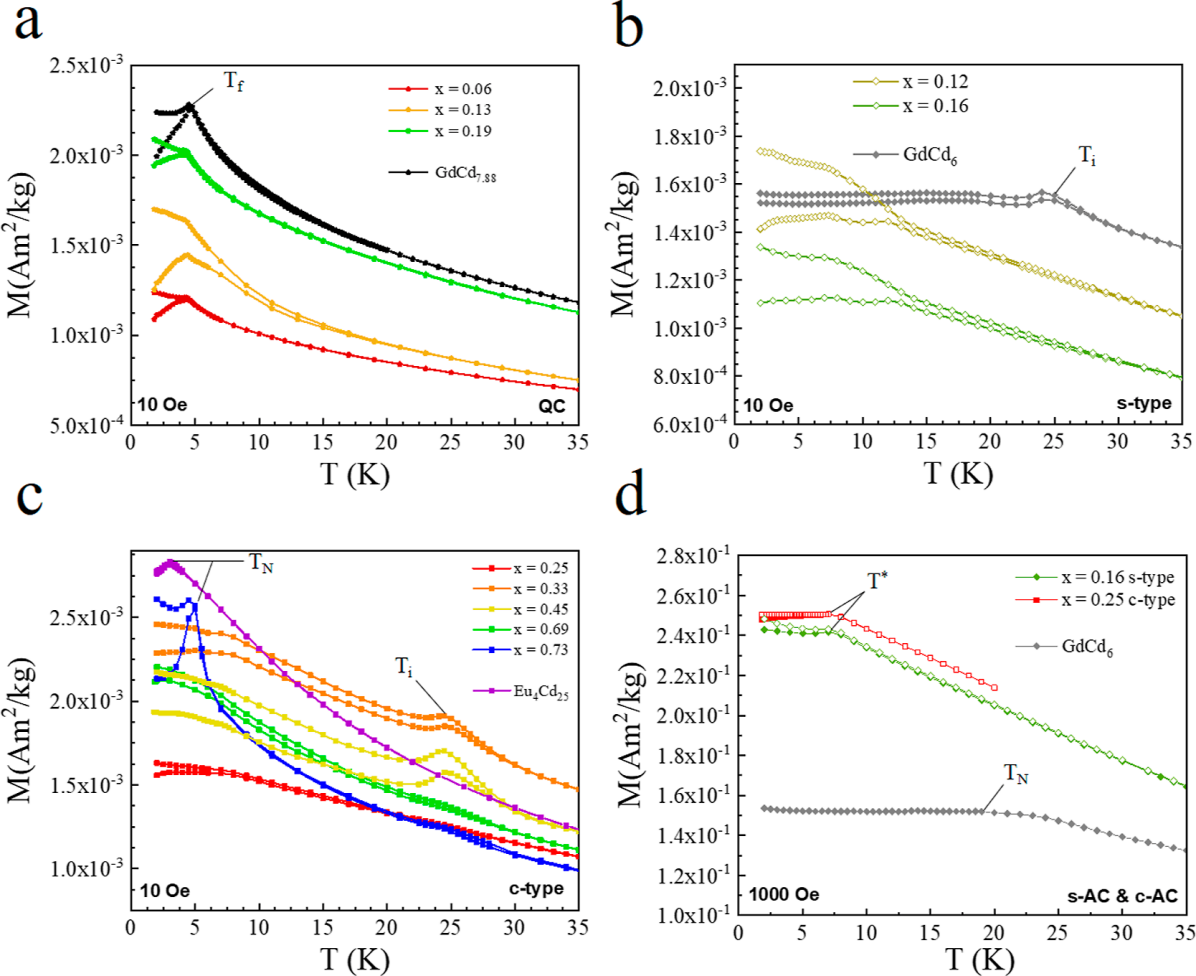
Zero-field-cooled and field-cooled magnetization plots of (a) QCs, (b) s-type 1/1 ACs compared to GdCd_6_, and (c) c-type 1/1 ACs compared to Eu_4_Cd_25_. (d) ZFC-FC plots under 1000 Oe for s- and c-type 1/1 ACs. We annotate *T*_f_ as the onset of glassy behavior in QCs, *T*_N_ as the antiferromagnetic transitions, T* as the onset of short-range-like transition, and *T*_i_ as the anomaly or instability near 26 K observed in the undoped GdCd_6_ and some c-ACs.

The magnetic behavior of all the quasicrystalline Gd_1–*x*_Eu_*x*_Cd_7.6±α_ samples was found to be similar to that of the binary i-GdCd_7.88_ QC,^26^ showing a spin glass freezing at low temperatures. The results are summarized in Figures 4a and S10 for memory experiments^26^ on the QCs with *x* = 0.06 and 0.19. The freezing temperature *T*_f_ is slightly lowered compared to that of the binary GdCd_7.88_ QC, which differs from Y dilution in the Gd–Cd QC^16^ where the substitution of Gd with nonmagnetic Y atoms led to a more striking lowering of *T*_f_ at similar doping concentrations. The undoped GdCd_7.88_ QC sample showed a freezing temperature *T*_f_ ∼ 4.7 K, which gets shifted to *T*_f_ ∼ 4.3 K in Eu-doped QC samples (*x* = 0.06, 0.13, and 0.19). As shown in the Supporting Information and Figure S7a,c, all the QCs show similar high field magnetic susceptibility curves and almost linear “S-shaped” hysteresis M(H) curves. The composition of the QC samples is also found to have a slightly decreased Cd content and is found to be closer to RCd_7.6±α_, with α ∼ 0.2. The exact mechanism by which the total Cd content is reduced is not known and would require a QC structural refinement, but this could indicate that Eu replaces Cd preferentially in some sites of the quasicrystalline structure, either by reducing the inherent chemical mixing on the icosahedral shell or on the double Friauf polyhedron (DFP) sites unique to QCs and 2/1 ACs. Previous QC structure refinements in i-R-Cd systems (R = Gd, Dy, Tm) showed that the three rare-earth sites existing in Tsai QCs, i.e., one for the icosahedral shell and two in the DFPs, were nonequivalent with respect to Cd dilution content for trivalent rare earth elements.^12^ Another possibility for the Cd depletion would be the replacement of the innermost tetrahedron of some Tsai clusters with a Eu ion, a structural change seen in other ternary Tsai ACs.^27^ In comparison, the magnetic behavior of the s-ACs was found to be similar to that of their Y-doped counterparts,^16^ without the apparent low-field anomaly in the magnetic susceptibility at *T*_i_ ∼ 26 K observed in the undoped 1/1 AC. That anomaly is, however, observed in the c-type 1/1 ACs to a varying extent, being more prominent for *x* = 0.33 and 0.45. We also note that the obtained Curie–Weiss temperature parameter θ_CW_ is found to be negative in all samples, reflecting the antiferromagnetic character of the magnetic interactions. The results of the Curie–Weiss analysis are given in the Supporting Information, Section II. Apart from the 1/1 ACs with *x* = 0.73 and Eu_4_Cd_25_, there is no clear evidence of (antiferro)magnetic transition in the data collected in 10 Oe presented in Figure 4b,c, even if magnetic irreversibility and magnetic anomalies are observed at low temperatures. At low doping, as seen in Figure 4d, a plateau similar to that marking the antiferromagnetic transition of the undoped GdCd_6_^4,16^ is observed in higher magnetic fields, albeit shifted to lower temperatures. This suggests a weakening of the antiferromagnetic interaction and a possibly short-ranged antiferromagnetic order below T* ∼ 10 K. This is consistent with the Curie–Weiss temperature θ_CW_, which decreases in absolute value as a function of *x* up to *x* = 0.25. The magnetic behavior is, however, not well defined for larger *x*, even if the θ_CW_ values for all ACs increase with *x* up to *x* = 0.45 and their high field susceptibility curves monotonically shift with *x* between that of GdCd_6_ and that of Eu_4_Cd_25_ (see Figure S7d). Note that the M(H) curves at *T* = 2 K are antiferromagnetic-like (i.e., linear with H) for all 1/1 AC samples (see Figure S7b).

To summarize, while the doped QCs retain the spin glass behavior of the parent compound, the ACs show predominantly antiferromagnetic interactions; however, it is not clear that all the Eu-doped crystals display long-range antiferromagnetic order akin to the end compounds GdCd_6_ and Eu_4_Cd_25_.

**Figure 5:**
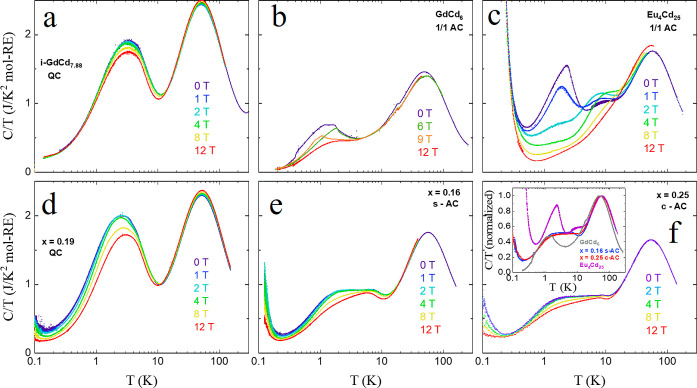
Specific heat (plotted as *C*/*T*) as a function of temperature for (a) the undoped GdCd_7.88_ and (d) *x* = 0.19 QCs. The corresponding plots of 1/1 ACs for (b) the undoped GdCd_6_ and (c) Eu_4_Cd_25_, (e) the s-type 1/1 AC with *x* = 0.16, and (f) the c-type 1/1 AC with *x* = 0.25 are also shown. The inset shows the normalized *C*/*T* data of the 1/1 ACs. The corresponding data for the QC with *x* = 0.06 and the c-AC with *x* = 0.45 is shown separately in Figure S11.

The specific heat of Eu-containing QC and AC samples was acquired in the temperature range of 0.1–200 K. The QC samples consisted of the undoped GdCd_7.88_ and the Eu-doped samples at concentrations *x* = 0.06 and 0.19. As for the 1/1 ACs, they consisted of GdCd_6_, Eu_4_Cd_25_, the s-ACs at concentration *x* = 0.16, and the c-ACs at concentrations *x* = 0.25 and *x* = 0.45. The results are summarized in Figure 5 and Supporting Information, Figure S11. All of the QC samples show a similar specific heat behavior, characterized by a broad peak in the vicinity of the spin freezing temperature. Upon close inspection, the broad peak feature is slightly shifted to a lower temperature and slightly broadened as the Eu concentration increases. At low temperatures, *C*/*T* decreases with an increasing magnetic field; the effect is more prominent for larger *x*. In the case of the 1/1 AC samples, the specific heat follows a more complex trend at low temperatures. The GdCd_6_ 1/1 AC exhibits a small but sharp onset at *T*_N_ = 19 K and a plateau between 1.1 and 1.7 K and then decreases at lower temperatures. At low Eu concentrations, the sharp onset and low-temperature features vanish, leaving only an inflection below *T* = 10 K and a broad plateau in the *C*/*T* plots. These inflections occur in the vicinity of T* and may reflect the short-ranged magnetic order of the doped ACs below that temperature (cf. Figure 4d). On the other hand, the c-AC at concentration *x* = 0.45 shows a *C*/*T* response larger than any other 1/1 AC investigated here, with an additional broad peak akin to that observed for QCs centered around ∼3 K (see Supporting Information, Figure S11). For that composition and for *x* = 0.33, the 26 K magnetic anomaly of the parent compound is observed more strongly. This may be related to the increase in the magnetic interactions suggested by |θ_CW_| increasing from *x* = 0.25 to *x* = 0.45. The specific heat of Eu_4_Cd_25_ was also investigated, with a steady increase in the specific heat starting around ∼16 K, incidentally matching the Curie–Weiss temperature of θ_CW_ = −16 K observed in this sample. The low-temperature peak onset also matches the Néel transition of *T*_N_ = 2.9 K from the magnetic measurements (see Figure S9). The specific heat field dependence of Eu_4_Cd_25_ is unusual for an antiferromagnet, with the low-temperature broad peak being almost entirely suppressed at only 2 T and a magnetic field-dependent broad peak centered around 7 K (see Figure 5c).

## Discussion

By comparing the Gd–Cd and Eu–Cd binary phase diagrams, we can notice that the 1/1 AC phase exists in both cases. In the Cd-rich region, a narrow window for the QC formation of GdCd_7.88_ exists in the former case and a wider region for the formation of tetragonal EuCd_11_ is present in the latter. As the Eu content in the starting composition rises, the liquidus line is gradually lowered (Figure 1b). The tetragonal RCd_11_ phase becomes achievable in the phase diagram as the starting composition Eu content increases, leading to a temperature region where the two phases (QC and tetragonal) coexist, as observed for rare-earth proportions R ≡ Eu_*y*_Gd_1–*y*_ with *y* = 0.4–0.55. Below a starting concentration of *y* = 0.4, only the quasicrystalline phase appeared to be present down to a centrifugation temperature of 335 °C. Within the range of *y* = 0.4–0.5, it is still possible to obtain large single grains by raising the centrifugation temperature to 360 °C, closer to the liquidus line. Syntheses in the region above *y* = 0.55 mainly showed the tetragonal phase, although microscopic QC crystallites could be observed from SEM imaging in samples with starting material concentrations of *y* = 0.6. The upper limit of the temperature where the tetragonal phase forms (increasing with Eu content) matches the liquidus line (decreasing with the Eu content) around *y* = 0.6, making QC formation impossible using the self-flux method above this value. This is supported by DSC data of the different phases synthesized (see Figure S3).

**Figure 6:**
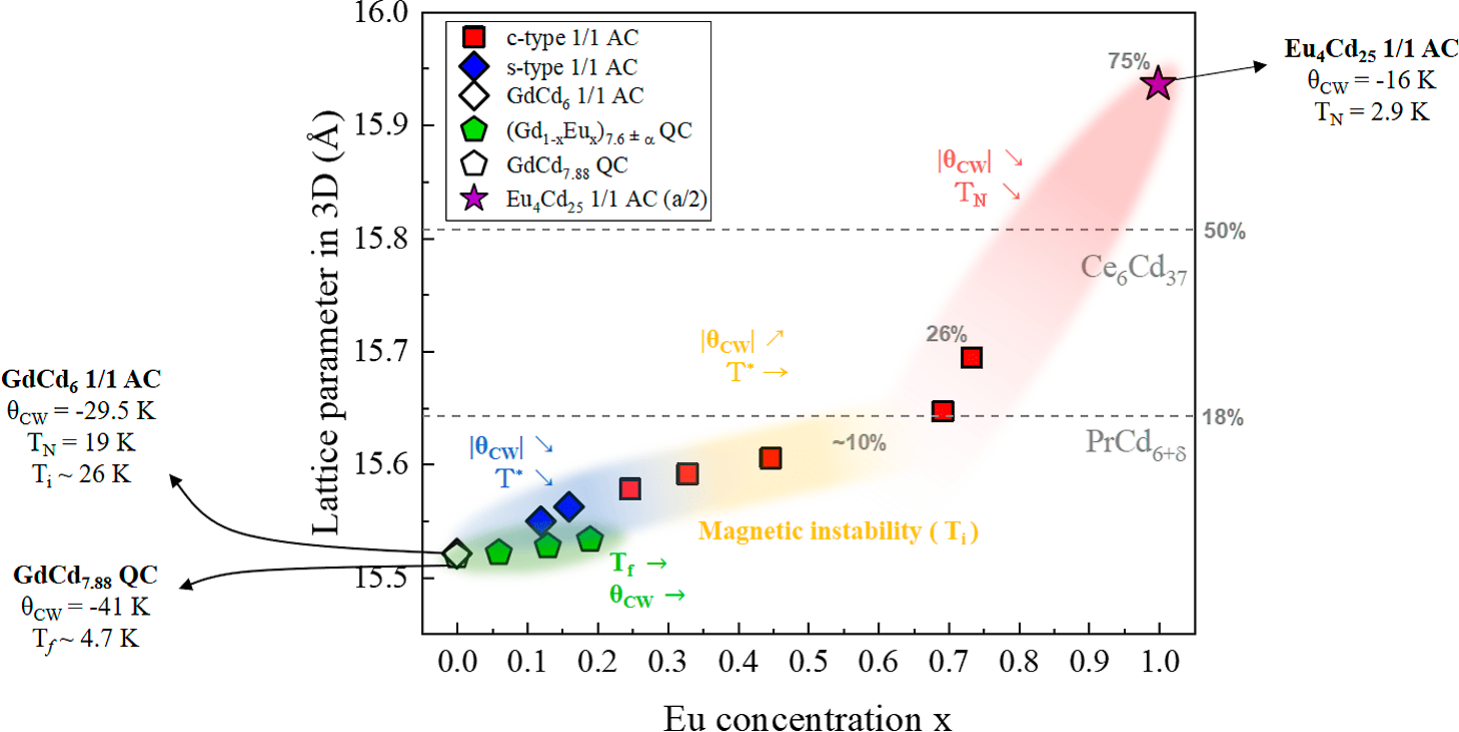
Lattice parameters of the Eu-doped 1/1 AC series and the *a*_1/1_ equivalents of the QC series. The lattice parameters of other 1/1 ACs with M6 site equivalents are shown with dashed lines, with partial occupancies marked in gray. The magnetic parameters for all samples are summarized in Table S4.

Europium, depending on its local crystalline environment, can be found either in its Eu^3+^ ion state, in its Eu^2+^, or in a mixed state, which includes both species. The magnetic properties depend strongly on the valence state. The Eu^3+^ represents an unusual case of magnetism where, in its ground state, the total angular momentum is zero (J = L–S = 0). Experimentally, compounds with only the Eu^3+^ present can show a nonzero magnetic moment described by Van Vleck paramagnetism if the temperature is large enough.^28^ In the case of both Eu^2+^ and Gd^3+^, however, the theoretical effective moment is 7.94 μ_B_ and is independent of temperature. Apart from the two s-type samples, the effective moment *p*_eff_ determined from Curie–Weiss analysis of all QCs and c-ACs is consistently found within about 3% of the theoretical value for Eu^2+^ and Gd^3+^ (see Table S4). The lower effective moment values observed for the s-ACs could reflect the partial Eu^3+^ character of Eu for those samples. Interestingly, the evolution of the magnetic curves upon doping for the s-type 1/1 ACs is similar to that of GdCd_6_ doped by nonmagnetic Y ions (vanishing of the anomaly at *T*_i_ = 26 K). In the case of the other ACs, we note that the magnetic behavior of the pure Eu_4_Cd_25_ 1/1 AC and the c-ACs is compatible with a full occupancy of Eu^2+^ ions. The samples with a mixture of Gd and Eu can be seen as similar to Eu_4_Cd_25_ but with added chemical pressure since their lattice parameters are contracted relative to the lattice parameter Eu_4_Cd_25_ would have from the *Im*3̅ perspective (see Figure 2). This may explain the Eu^3+^ character of the Eu-doped samples with the lowest lattice parameters. Valence fluctuation has been studied for Yb using direct pressure in the YbCd_5.7_ QC^9^ and Yb–Au–Zn ACs,^29^ as well as chemical pressure in Yb–Au–Al QCs.^30^ Note that the relation between valence and magnetic behavior is reversed between Yb and Eu, i.e., Eu^3+^ and Yb^2+^ are expected to be nonmagnetic at low temperatures, while Eu^2+^ and Yb^3+^ both possess a stable magnetic moment. While there is no investigation of the impact of pressure on Eu-containing ACs, it is reasonable to assume that physical or chemical pressure could affect the effective valence of Eu^31^ in a similar manner as the lattice parameter gets closer to the undoped GdCd_6_. When comparing the Eu doping in the c-ACs to doping with nonmagnetic Y atoms similar in size to Gd, there is a less significant decrease in the transition temperature and |θ_CW_|, i.e., the magnetic interaction remains significant. The same applies to the QC samples.^16^Figure 6 summarizes the evolution of the structural and magnetic properties of the QCs and ACs as a function of Eu doping.

**Figure 7:**
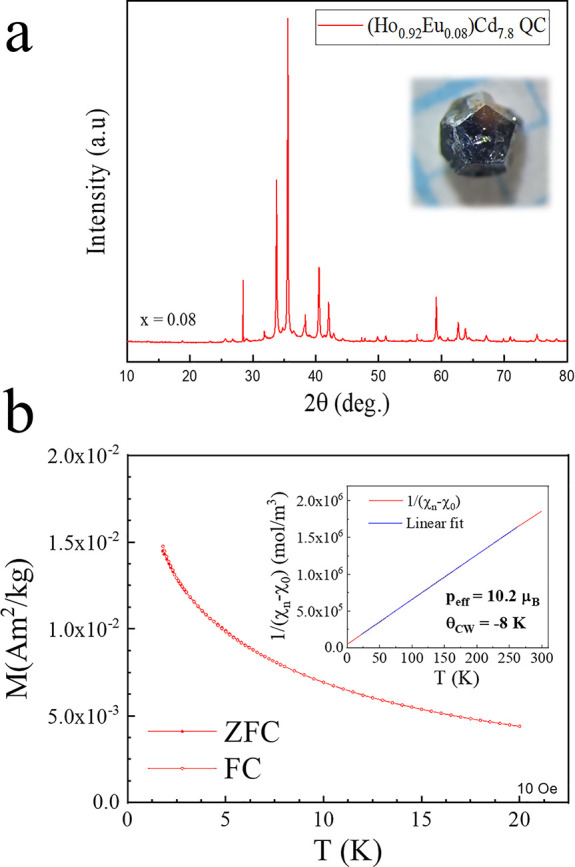
(a) PXRD pattern of a synthesized Eu-doped Ho–Cd QC; the inset shows the typical pentagonal facets of the single grains. (b) Magnetic measurement data of the dc magnetization in ZFC and FC under 10 Oe. No transition is observed down to the measurement system limit of 2 K. The inset shows the Curie–Weiss fit of the inverse susceptibility with the measured parameters *p*_eff_ and θ_CW_.

Synthesis with Eu-doping can be generalized to other rare earth R–Cd systems. One advantage of choosing Gd and Eu as the magnetic elements is that Eu^2+^ and Gd^3+^ should be magnetically very similar, but if the limiting factor explaining the absence of Eu-based QC were to be the size of Eu^2+^, as is often speculated, doping in a quasicrystalline system with a smaller lattice parameter should allow more Eu to be included in the structure before the effective lattice parameter becomes too large for QCs to form, and the size-limited saturation should appear at a similar hyperlattice parameter *a*_6D_. To test this hypothesis, we have synthesized a Eu-doped Ho–Cd QC sample (see Figure 7). The (Ho_0.92_Eu_0.08_)Cd_7.8_ QC crystal synthesized was found to have a larger Cd content than the undoped HoCd_7.6_ QC.^32^ The Eu concentration, however, shows a similar behavior compared to the Eu-doped Gd–Cd QCs, with a final Eu concentration (*x* = 0.08) significantly below the nominal starting material concentration (*y* = 0.4). In comparison, at identical starting concentration y, the final concentration achieved was *x* = 0.13 in Eu-doped Gd–Cd QCs. This large discrepancy between starting material and final Eu concentration could indicate that the limiting factor preventing the formation of Eu-containing QCs is not the size, especially since the hyperlattice parameter of the YbCd_5.7_ QC, *a*_6D_ = 8.045 Å,^33^ is much larger when compared to the values in our Gd_*x*_Eu_1–*x*_Cd_7.6±α_ QC samples, ranging from *a*_6D_ = 7.972 Å to *a*_6D_ = 7.980 Å, with the limits given by the undoped GdCd_7.88_ and the Eu-doped QC at *x* = 0.19, respectively. The hyperlattice parameter of the Eu-doped Ho–Cd QC with *x* = 0.08 is found to be even lower than those of the aforementioned samples at *a*_6D_ = 7.944 Å. If quasicrystalline growth was limited only by the rare-earth average size, one would expect the lattice parameter at saturation to be similar to the largest known *a*_6D_ from Cd-based samples, e.g., CaCd_5.7_^8^ or Yb–Cd–Mg QCs,^34^ with *a*_6D_ = 8.105 Å and *a*_6D_ = 8.144 Å, respectively.

A more compelling criterion than size that can explain the destabilization of the quasicrystalline phase appears to be the occupancy of the M6 site mentioned throughout this study. In addition to Eu_4_Cd_25_, the M6 sites have been observed to be partially occupied for other binary R–Cd systems: in PrCd_6+δ_,^24^ with space group *Im*3̅, and in Ce_6_Cd_37_,^35^ with space group *Pn*3̅, for which half of the M6 sites (four per unit cell) are occupied in a periodic manner, forming a tetrahedron which breaks the inversion center symmetry without doubling of the unit cell. The M6 site corresponds to Wyckoff site 4d in that space group. Note that the M6-equivalent site occupancy is 18% for PrCd_6+δ_, 50% for Ce_6_Cd_37_, and 75% for Eu_4_Cd_25_, as indicated in Figure 6. In the c-AC samples, the M6 occupancy appears to plateau around 10% occupancy up to *x* = 0.7 and then rises abruptly above it. The highest M6 occupancy considered in this work is ∼25%, at Eu concentration *x* = 0.73. The increase in the lattice parameter in the region 0 < *x* < 0.69 is attributed mainly to the larger ionic size of Eu^2+^ compared to that of Gd^3+^ but is expected to rise more sharply in the region 0.69 < *x* < 1 as the occupancy of the M6 sites by Cd starts to increase further. The 1/1 AC equivalent lattice parameters *a*_1/1_ of the QC samples synthesized do not expand as much as the s-AC samples at similar concentrations.

We find that all of the known stable Tsai-type QC systems, such as the often studied R–Cd and R–Cd–Mg (R = Y, Ca, Gd–Lu), Sc–Zn, Sc–Cu–Zn, and Sc–Zn–Mg have, when reported, either low or no occupancy on the M6 site of their 1/1 AC counterparts (less than 7%; see Table 3). On the other hand, all reported metastable, fast-quenched quasicrystalline samples show a large M6 site occupancy in their 1/1 ACs when SCXRD refinement is available (from 37 to 100%). Likewise, for the compounds without a known QC phase, occupancies of 14–100% have been reported. We observe only one exception to the rule, with a recently reported Yb–Au–Zn 1/1 AC, a system which shows no occupancy of the M6 site and yet possesses only a metastable QC phase. This could, however, be explained by another significant structural change specific to this sample since, for this material, a large fraction (51%) of the Tsai cluster’s innermost tetrahedron (referred to as position M1 in this work) is substituted by an Yb atom at Wyckoff site 2a. These findings are summarized in Table 3. Note that an elongation of the electron density along its 3-fold axis often appears at the M6 site, leading to a slight shift in position which may require one to consider the cube interstice site as a Wyckoff position 16f for better refinements. If a stable QC exists for a specific system, it appears that its 1/1 AC counterpart possesses no significant structural change from the prototypical Tsai cluster, i.e., no interstitial site between the icosidodecahedron and the rhombic triacontahedron, as well as only the tetrahedron as their innermost shell.

**Table 3:** Examples of the M6 Site Occupancy, with the Existence and Stability of a QC Phase for Various 1/1 AC Compounds^b^

1/1 AC system	QC existence	M6 site occupancy	center RE	ref
R–Cd	yes	0		(24)
Yb–Cd–Mg	yes	0.069^a^	0.005	(34)
Sc–Zn–Mg	yes	0		(37)
Sc–Cu–Zn	yes	0		(38)
Yb–Au–Al	metastable	0.92		(6)
Ca–Au–In	metastable	0.47		(39)
Tb–Au–Ga	metastable	1	0.026	(40)
R–Ag–In	metastable	0.37 (R = Eu)		(41),^42^
Yb(Zn,Al)	no	1	0.014–0.016	(43)
Yb–Au–Ga	no	1		(44)
Gd–Au–Al	no	1		(45)
Gd–Au–Ge	no	0.37	0.1	(46)
Yb–Au–Ge	no	0.34	1	(46)
Gd–Au–Si	no	0.14		(46)
Yb–Au–Zn	metastable	0	0.51	(29)

a Occupancy refined as the position 16f due to the elongation of the electron density along the 3-fold axis.

b When applicable, the rate of substitution of the inner tetrahedron by a rare-earth atom (Wyckoff site 2a) is also mentioned. The QC stability information was obtained from ref^5,36^

One important system of stable QC for which no 1/1 AC counterpart SCXRD refinement has been reported is the M–Ag–In system (M = Yb, Ca).^8^ In that case, interestingly, stable QCs can be obtained with Yb or Ca, but only metastable QCs have been reported for other rare earth magnetic elements M = Pr–Tm.^42^ The interstitial occupancy along the 3-fold Tsai cluster attachments (c-linkages) can also occur in 2/1 approximants, with seven c-linkages (i.e., cube interstitial sites) connected to each Tsai cluster instead of eight in the case of 1/1 ACs,^47^ so the present criterion could be expanded to 2/1 ACs when the phase is achievable.

Since high pressure can be used to reduce the lattice parameter, the present work could motivate expanding the use of high-pressure synthesis to Tsai-type QCs; other types of icosahedral systems (e.g., Al–Cu–Fe) have been shown to be stabilized by pressure.^48^ More research is needed to gain more certainty on the relation between the M6 site occupancy, inner tetrahedron substitution, and the destabilization of quasicrystalline phases. Nonetheless, along with the *e*/*a* average electron valence ratio, these new occupancy criteria could be used to narrow down the list of candidate systems, facilitating the discovery of new, stable QCs from a systematic, careful analysis of the existing 1/1 AC phases.

## Conclusions

In conclusion, the addition of Eu in the Gd–Cd AC and QC systems leads to very different outcomes depending on whether the lattice is periodic or quasiperiodic. We discovered a new type of 1/1 AC, referred to as c-type, for a large enough Eu concentration. The c-type 1/1 ACs present cubic single-crystal facets, with the same point group as GdCd_6_, but extra interstitial Cd sites located between the icosidodecahedron and rhombic triacontahedron shells of the Tsai cluster. This phase possesses more chemical disorder, with the large Eu atoms causing extra Cd to fit into the structure. As opposed to the standard-type 1/1 AC, any Eu concentration can be achieved for this variant, and the starting material compositions are found to be consistent with the final compositions. On the other hand, the standard-type 1/1 approximant phase, referred to as s-type, can be selected by reducing the final annealing temperature. This phase shows a saturation point for Eu at 16% of the rare earth magnetic elements, similar to the saturation of 19% found for the QCs. The quasicrystalline samples, however, show a less significant expansion of the Tsai cluster diameter when compared to the effect of Eu doping on the lattice parameter of the 1/1 AC samples. In terms of magnetism, the QCs are only mildly affected by the Eu doping, but the 1/1 AC magnetization shows a more complex behavior. The Eu doping can be applied to other rare-earth QCs and ACs, as we have shown here with the synthesis of an Eu-doped (Ho_0.92_Eu_0.08_)Cd_7.8_ QC. The saturation behavior observed is inconsistent with the idea of the average rare-earth size being the limiting factor for QC formation. Instead, we observe that the concentration *x* at which the Eu saturates in the Gd–Cd QC system appears to coincide with the increase in the Wyckoff 8c site occupancy in the 1/1 ACs to a nonzero value. This suggests that the 1/1 AC counterpart of stable QCs never possesses more than ∼7% occupancy of the 8c site, hereby offering a new criterion for the stability of Tsai-type QCs.

## Supporting Information Available

Synthesis details, additional magnetic data, and additional specific heat capacity data (PDF)
