# Supplementary material for: Eu Doping in the GdCd7.88 Quasicrystal and Its Approximant Crystal GdCd6
Source: Inorg Chem. 2024 Mar 1;63(11):5040–51. doi: 10.1021/acs.inorgchem.3c04500 (PMC10951946; doi:10.1021/acs.inorgchem.3c04500)
Supplement: Supplementary file 1 — ic3c04500_si_001.pdf [file ic3c04500_si_001.pdf]

Supplementary information

Eu doping in the $GdCd_{7.88}$ quasicrystal and its approximant crystal $GdCd_6$

Fernand Denoel[1†*], Yu-Chin Huang[2†], Neha Kondedan[3], Andreas Rydh[3], Cesar Pay-Gómez[2], and Roland Mathieu[1*]

1 Department of Materials Science and Engineering, Uppsala University, Box 35, 751 03, Uppsala, Sweden
2 Department of Chemistry-Ångström laboratory, Uppsala University, 751 21 Uppsala, Sweden
3 Department of Physics, Stockholm University, 10691, Stockholm, Sweden

* *fernand.denoel@angstrom.uu.se; roland.mathieu@angstrom.uu.se*

Table of contents

I) Synthesis and phases characterization

a) **Synthesis comments, EDX and ICP results**
b) **DSC results and phase diagram**
c) **Structure**

II) Additional magnetic data

III) Additional heat capacity data

† Authors contributed equally

I) Synthesis and phases

a) Synthesis comments, EDX and ICP results

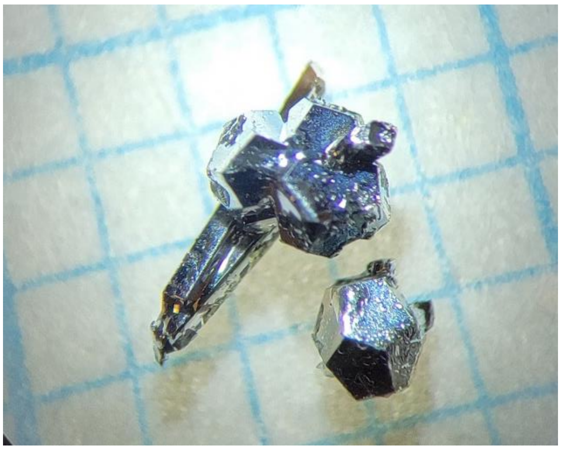

Figure S1: Picture of single crystal grains from the batch with starting materials composition $(Gd_{0.5}Eu_{05})_{0.8}Cd_{99.2}$ centrifuged at 335 °C. Both tetragonal grains (with long rectangular prism shape) and quasicrystalline (dodecahedron-shaped) are present after centrifugation.

Quasicrystal and tetragonal crystals were formed using the self-flux method. They were observed to crystallize simultaneously if the final annealing temperature was set below a temperature-dependent threshold, which raises with the Eu concentration x to meet with the liquidus line above x = 0.55. As the Eu concentration raises, the temperature window for which only QCs are obtained after centrifugation becomes narrows, making it nearly impossible to obtain macroscopic grains from centrifugation above x ~ 0.5. In the pure Gd-Cd phase diagram, quasicrystals can only be obtained in the Gd-poor region, up to ~1% Gd starting material. Above that threshold, crystals will form as 1/1 ACs. Adding Europium does not appear to shift this limit towards a higher rare earth (RE) percentage, as evidenced by the batch with starting material $(Gd_{0.4}Eu_{0.6})_{1.2}Cd_{98.8}$, which showed only s-type 1/1 ACs at 1.2% RE. The Cd per rare-earth ratio was estimated from SCXRD refinements, and the Gd/Eu ratio were established with EDX data for the 1/1 ACs and ICP for the QCs. The reason why ICP was chosen is the lower concentration of some of the samples, along with the lower at% concentration of QCs compared to 1/1 ACs, making the EDX method unreliable.

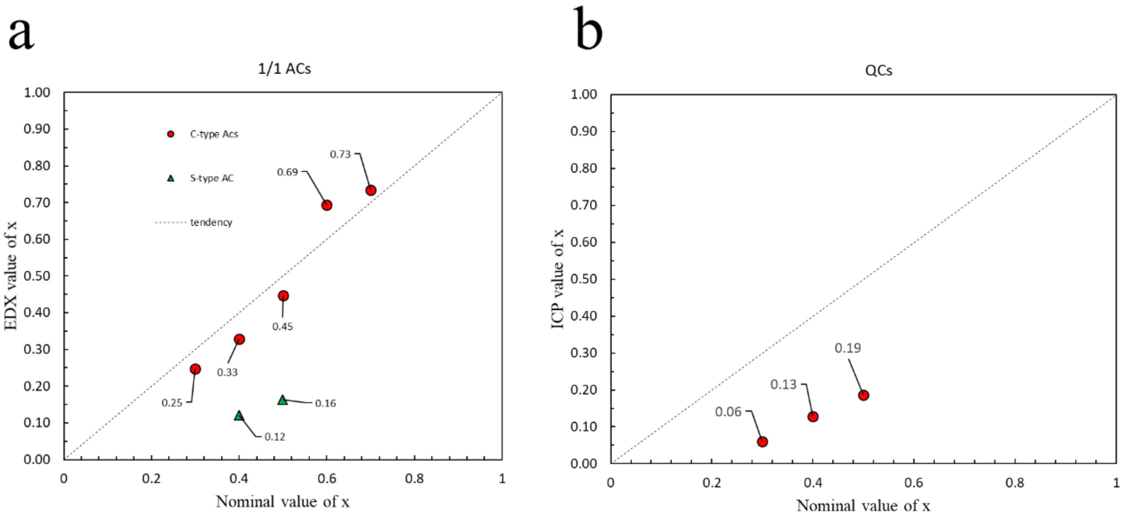

Figure S2: Proportion of Eu atom in the structure of (a) c-type and s-type 1/1 ACs and (b) quasicrystals, compared to the nominal value.

Table S1: Summary of the syntheses conditions and crystalline phases obtained.

**Nominal comp.**	**Slow cooling temp. Start /end T**	**Centrifug. temp. (°C)**	**Phase**	**Crystal facets**
**$(Gd_{0.7}Eu_{0.3})_{0.8}Cd_{99.2}$**	1°C/ hour; 455-335°C	335	QC	pentagonal
**$(Gd_{0.6}Eu_{0.4})_{0.8}Cd_{99.2}$**	1 °C/ hour; 455-335°C	335	QC+tetra	mixture
**$(Gd_{0.5}Eu_{05})_{0.8}Cd_{99.2}$**	1°C/ hour; 455-335°C	335	QC+tetra	mixture
**$(Gd_{0.5}Eu_{05})_{0.8}Cd_{99.2}$**	1°C/ hour; 480-360°C	360	QC	pentagonal
**$(Gd_{0.7}Eu_{0.3})_{10}Cd_{90}$**	2°C/ hour; 650-554°C	554	AC	cube
**$(Gd_{0.6}Eu_{0.4})_{10}Cd_{90}$**	2°C/ hour; 650-554°C	554	AC	cube
**$(Gd_{0.6}Eu_{0.4})_{z}Cd_{100-z}$***	2°C/ hour; 620-520°C	520	AC	cube
**$(Gd_{0.6}Eu_{0.4})_{u}Cd_{100-u}$***	2°C/ hour; 605-505°C	505	AC	cube
**$(Gd_{0.5}Eu_{0.5})_{10}Cd_{90}$**	2°C/ hour; 650-554°C	554	AC	cube
**$(Gd_{0.4}Eu_{0.6})_{10}Cd_{90}$**	2°C/ hour; 650-554°C	554	AC	cube
**$(Gd_{0.3}Eu_{0.7})_{10}Cd_{90}$**	2°C/ hour; 650-554°C	554	AC	cube
**$(Gd_{0.6}Eu_{0.4})_{2}Cd_{98}$**	2°C/ hour; 490-390°C	390	AC	standard
**$(Gd_{0.5}Eu_{0.5})_{2}Cd_{98}$**	2°C/ hour; 600-460°C	460	AC	standard

*** z = 3, 4, 5 ; u = 5, 6, 7, 8**

Table S2:EDX data of the 1/1 ACs.

**Centrifugation Temp.**	**Starting Gd:Eu ratio**	**Starting Eu (y)**	**EDX data**		**1/1 ACs**
			**Eu (at%)**	**Gd (at%)**	**Eu per f.u. (x)**
**554 °C**	**0.7:0.3**	0.3	3.45	10.52	0.25
	**0.6:0.4**	0.4	4.61	9.39	0.33
	**0.5:0.5**	0.5	6.27	7.73	0.45
	**0.4:0.6**	0.6	9.70	4.30	0.69
	**0.3:0.7**	0.7	10.28	3.72	0.73
**460 °C**	**0.7:0.3**	0.3	1.60	11.68	0.12
**390 °C**	**0.6:0.4**	0.4	2.30	11.87	0.16

Table S3: ICP data of the QCs.

**Centrifugation Temp.**	**Starting Gd:Eu ratio**	**Starting Eu (y)**	**ICP data**		**QCs**
			**Eu (at%)**	**Gd (at%)**	**Eu per f.u. (x)**
**335 °C**	**0.7:0.3**	0.3	0.73	11.27	0.06
	**0.6:0.4**	0.4	1.55	10.45	0.13
	**0.5:0.5**	0.5	2.24	9.76	0.19

b) DSC results and phase diagram

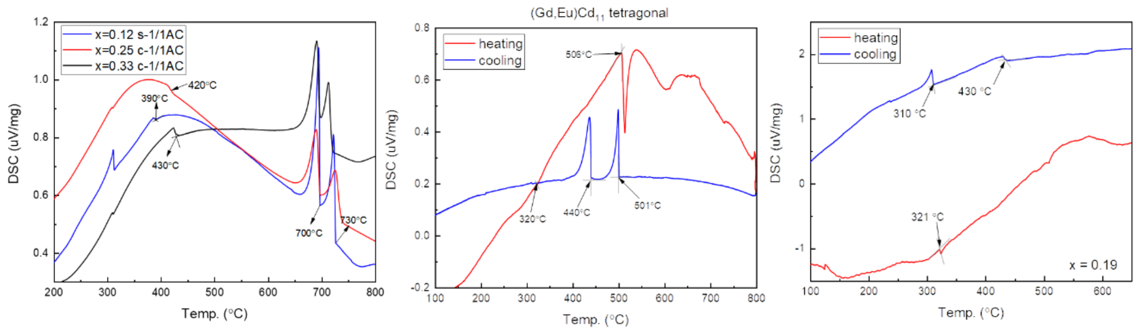

Figure S3: (From left to right) DSC measurements of the 1/1 ACs, tetragonal phase sample and a Eu-doped QC sample.

We investigated the three phases, 1/1 ACs, tetragonal and QC with DSC. In the 1/1 ACs, the formation temperature of the QC increases as Eu concentration increases from 390°C to 430°C. This observation is consistent with previous investigations and the QC single crystal DSC result. The QC with the highest Eu concentration showed a similar result, which indicates the upper limit of the formation temperature for Eu-doped QC is around 430°C. In the DSC result of the (Gd, Eu)Cd_{11} sample, we observed two formation temperatures, 440 and 501°C. According to the previous report, we expect the peak at 501 °C to be a consequence of the (Gd, Eu)Cd_{11} tetragonal phase formation, whereas the peak at 440 °C to should be linked to the QC phase. The summarized DSC results provided gives a possible answer as to why no QC in the binary Eu-Cd system appears achievable. For concentrations of Eu above x=0.25 in the ($Gd_{1-x}Eu_x$)Cd compounds, the QC and tetragonal phases coexist, and it is difficult to isolate the pure ($Gd_{1-x}Eu_x$)Cd QC phase with solution grown method to target the shrunk window.

c) Structure

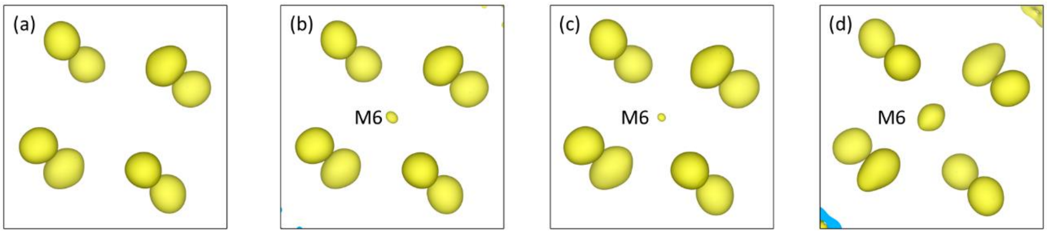

Figure S4: Cube interstice electron density isosurfaces surrounding the M6 position in the c-type 1/1 ACs at concentration (a) x = 0.25, (b) x = 0.45, (c) x = 0.69 and (d) x = 0.73.

The series of the Eu-doped 1/1 ACs present the electron density isosurfaces at the 15 -e/Å^3 level in the location of the M6 site, i.e., cube interstitial position. In Figure S4, (a) to (d), the electron density map has been generated from F_{obs} data of the individual single crystal diffraction dataset with the selected area. The Eu concentration increases in order, as x=0.25, 0.45, 0.69, 0.73 in the (a) through (d), as well as increasing the occupancy of the M6 site, respectively. From (a)-(d), the M6 site occupancy increase from left to right is 1.3%, 8%, 9.8%, and 25.6%, respectively. Although the additional Cd position is not visible at the electron density isosurface at the 15 e/Å^3 level in the x = 0.25 sample, it is visible at the electron density isosurface at the 8 e/Å^3 level. Therefore, we still take its ~1.3% occupancy into account, which is higher than the uncertainty value of 0.6%, and use it as a comparison with other samples.

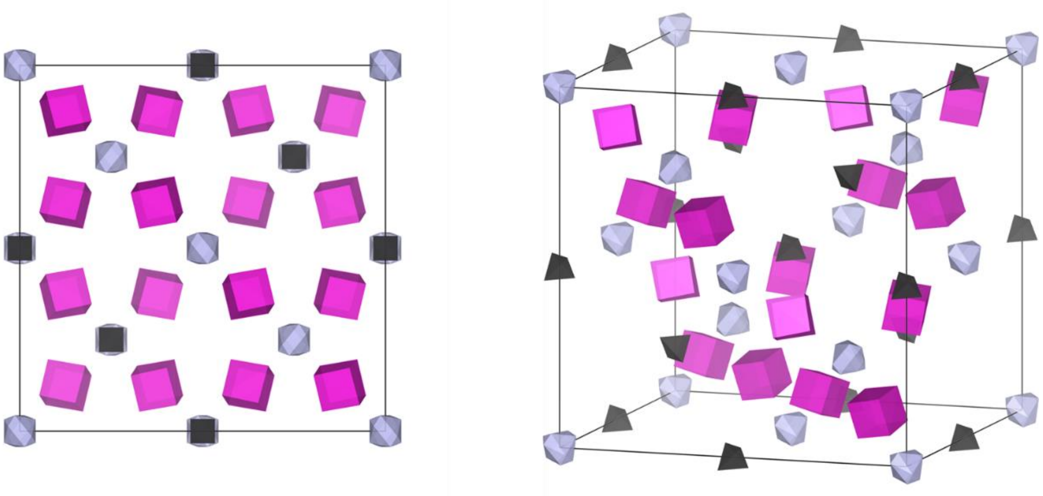

Figure S5: Unit cell of the Eu_4Cd_{25} 1/1 AC. The black tetrahedra are ordered while the light grey ones are random within the structure. All M6 sites equivalent within the structure are occupied except for the location of the 16 empty magenta cubes.

In total, there are 8 × 8 = 64 M6 equivalent sites in the Eu_4Cd_{25} structure, of which 16 are unoccupied. They are shown in the previous figure, by drawing the empty cubes given by 2 dodecahedron shell Cd positions along the (1 1 1) direction and equivalent, as well as 6 positions from the icosidodecahedra. The vacancies line up the edges of a large tetrahedron. In

total, there is 75% occupancy of the M6 equivalent (Wyckoff 8c) sites in the structure (64-16 out of 64). The face-centered structure can be understood as alternating ordered-disordered tetrahedra separated by half-step of the lattice in any direction (a, b or c) superposed with the rule giving the above vacancy tetrahedron.

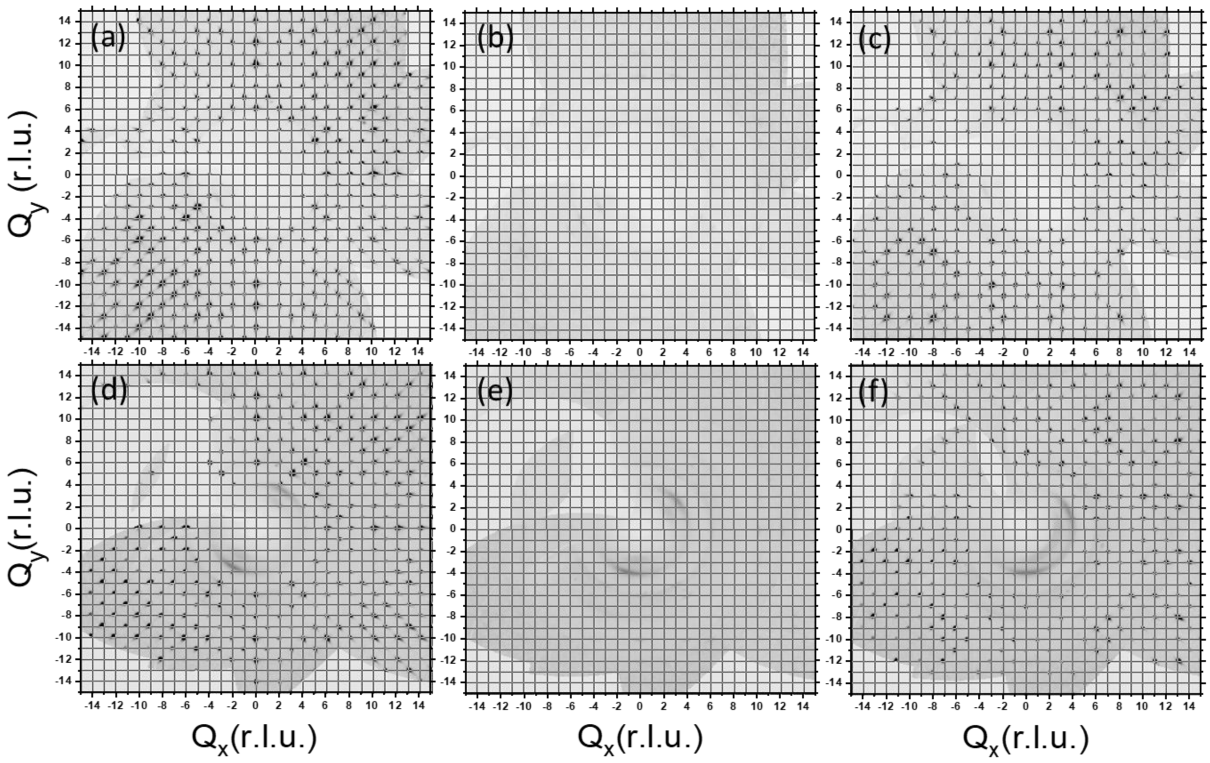

Figure S6: Reciprocal space of the c-AC with x = 0.45 at (a-c) 293 K and (d-f) 100 K. Reciprocal space slice of (a,d) **h k 0**, *(b,e)* **h k 0.5** *and (c,f)* **h k 1**.

II) Additional magnetic data

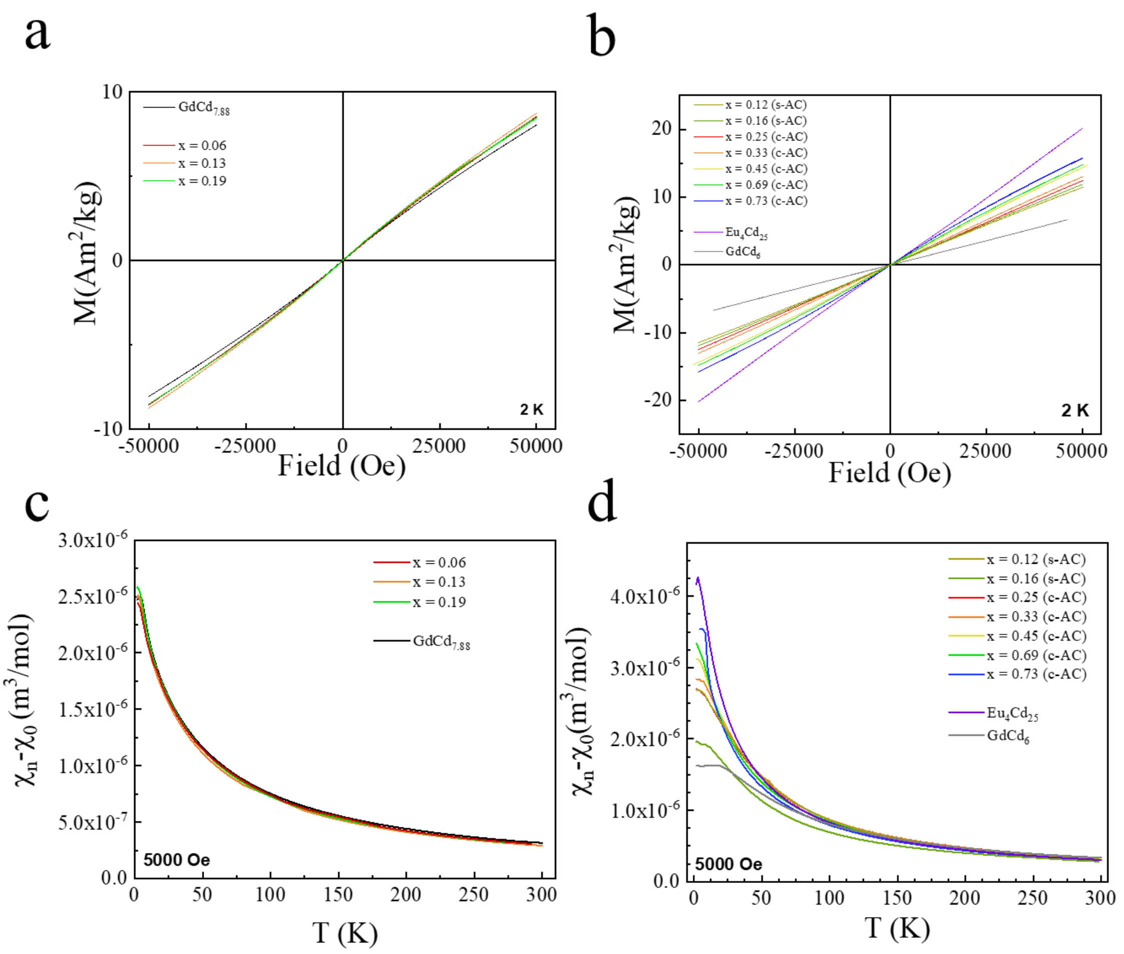

Figure S7: Magnetization as a function of field recorded at T = 2 K for the Eu-doped (a) QCs and (b)1/1 ACs. The magnetic susceptibility plots recorded under a magnetic field of 5000 Oe are presented in (c) for the QCs and (d) for the 1/1 ACs.

The results of the Curie-Weiss analysis are summarized below. The Curie Weiss temperature parameter **θ_{CW}** and effective moment **p_{eff}** were obtained from a linear fit of the inverse susceptibility, considering $\chi(T) = \frac{C}{T-\theta_{CW}} + \chi_0$ in the high temperature region.

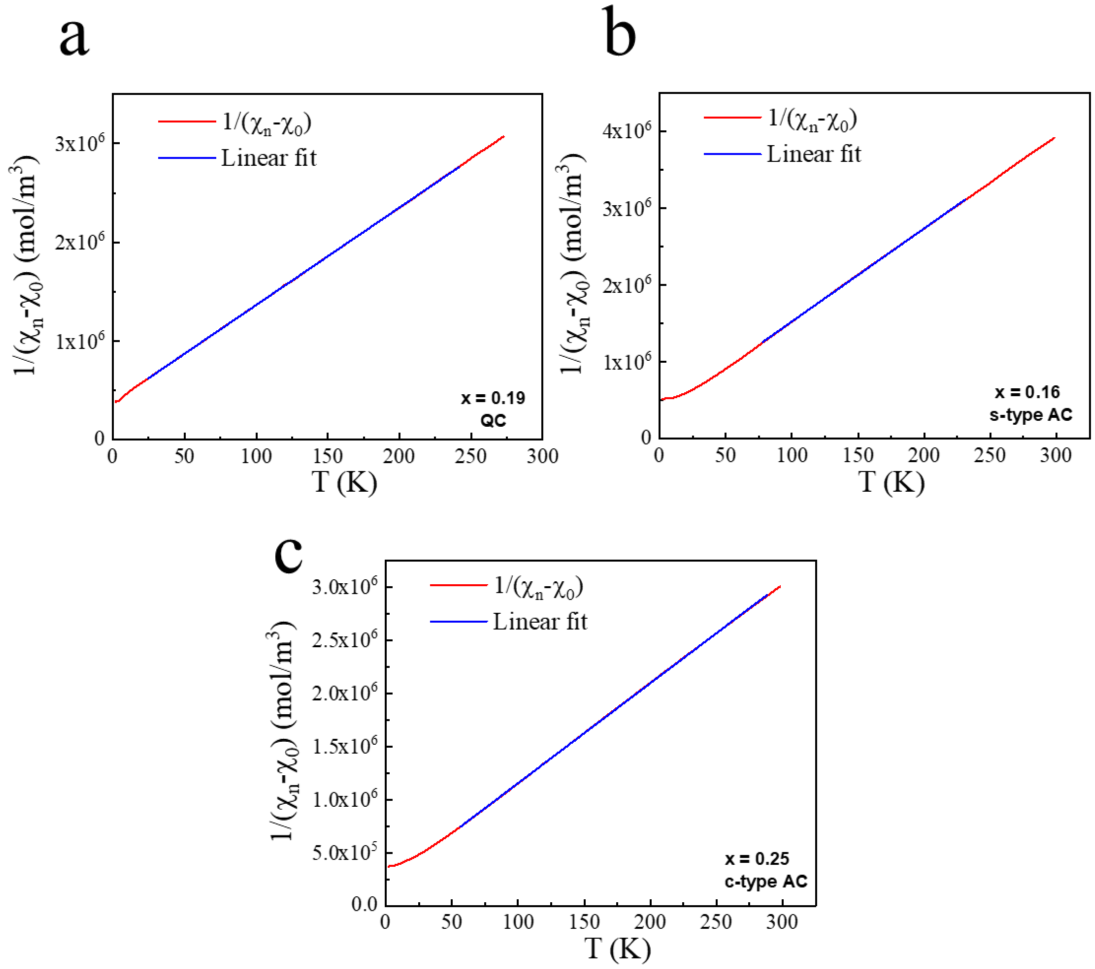

Figure S8: Examples of Curie-Weiss analyses. Inverse magnetic susceptibility plots of (a) the Eu-doped QC with concentration x = 0.19, (b) the s-type 1/1 AC with x = 0.16 and (c) c-type 1/1 AC with x = 0.25. The blue line are linear fits to the data.

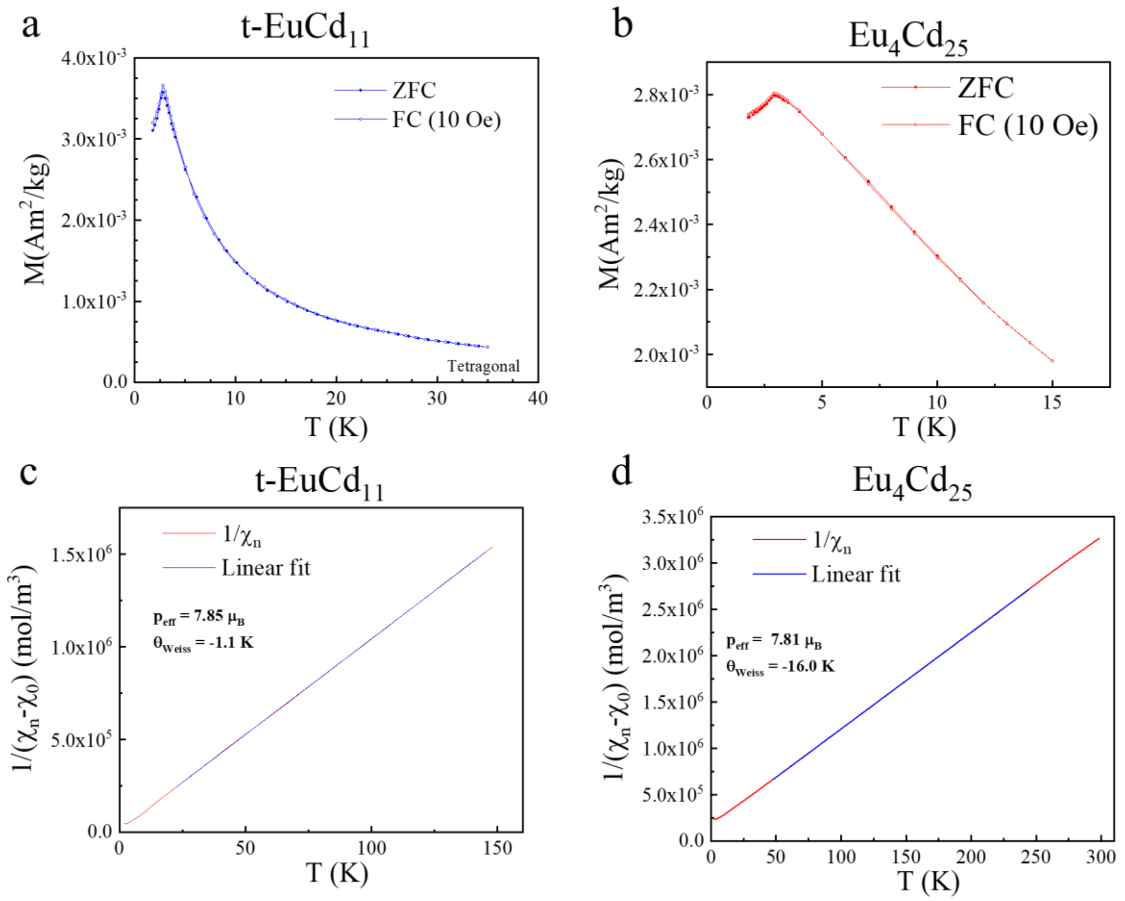

Figure S9: Zero field cooled and field cooled magnetization of (a) the tetragonal $EuCd_{11}$ phase and (b) The Eu_4Cd_{25} 1/1 AC. (c) Curie-Weiss fits of the inverse susceptibility of $EuCd_{11}$ and (d) The Eu_4Cd_{25} 1/1 AC.

Table S4: Summary of magnetic properties.

**Sample composition**	**Type**	**Eu (x)**	**θ_{CW} (K)**	**p_{eff} (μ_B)**	**χ_0 (m^3/mol)**
**$GdCd_6$**	s-AC	0	-29.5	8.07	-1.79e-09
**$Gd_{0.88}Eu_{0.12}Cd_6$**	s-AC	0.12	-26.2	7.65	-2.95e-09
**$Gd_{0.84}Eu_{0.16}Cd_6$**	s-AC	0.16	-20.6	7.01	+1.17e-08
**$Gd_{0.75}Eu_{0.25}Cd_{6.004}$**	c-AC	0.25	-16.4	7.9	-2.40e-08
**$Gd_{0.67}Eu_{0.33}Cd_{6.012}$**	c-AC	0.33	-19.6	8.11	-6.3e-8
**$Gd_{0.55}Eu_{0.45}Cd_{6.027}$**	c-AC	0.45	-26.1	8.11	-1.97e-08
**$Gd_{0.31}Eu_{0.69}Cd_{6.033}$**	c-AC	0.69	-24.1	7.96	-1.53e-08
**$Gd_{0.27}Eu_{0.73}Cd_{6.085}$**	c-AC	0.73	-20.7	7.77	-3.89e-08
**Eu_4Cd_{25}**	AC*	1	-16.0	7.81	-1.67e-08
**$GdCd_{7.88}$**	QC	0	-41	8.2	-8.30e-08
**$Gd_{0.94}Eu_{0.06}Cd_{7.62}$**	QC	0.06	-41.5	8.05	-2.75e-08
**$Gd_{0.87}Eu_{0.13}Cd_{7.4}$**	QC	0.13	-38.6	7.92	-1.5e-08
**$Gd_{0.81}Eu_{0.19}Cd_{7.54}$**	QC	0.19	-37.6	7.99	-1.15e-07

**superstructure*

The zero-field cooled (ZFC) and field-cooled (FC) magnetization measurements were performed under a field applied of 10 Oe for both the $EuCd_{11}$ tetragonal phase sample and the Eu_4Cd_{25} 1/1 AC. The magnetic susceptibility data was acquired up to larger temperatures under a field of 5000 Oe for both samples.

The relatively large Curie-Weiss temperature θ_{CW} = -16 K found for the Eu_4Cd_{25} compared to the observed Néel transition at T_N = 2.9 K indicates a frustrated antiferromagnet behavior.

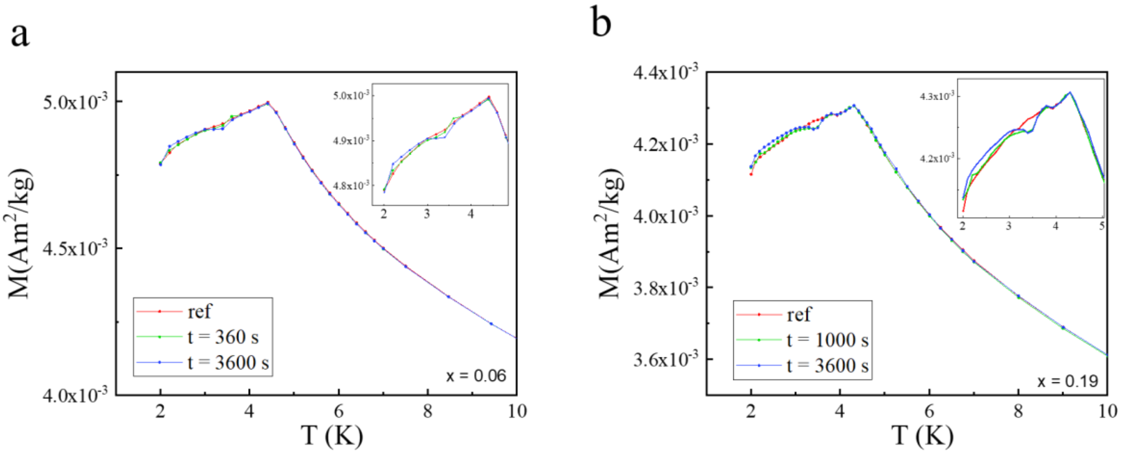

Figure S10: Magnetic memory measurement for the QCs at x = 0.06 and x = 0.19. an increase in waiting time at T_w = 3.5 K during cooling increases the magnetization difference in the ZFC plot around T_w.

Regarding the quasicrystalline Eu-doped samples. Memory experiments were performed on the samples at doping concentration x = 0.06 and x = 0.19. The samples were cooled down under no field applied and set to wait at temperature T_w = 3.5 K below their spin freezing temperature. As expected, depending on the waiting time, a difference of magnetization appears in the ZFC data when compared to the reference acquired without stopping step.

III) Additional Heat capacity data

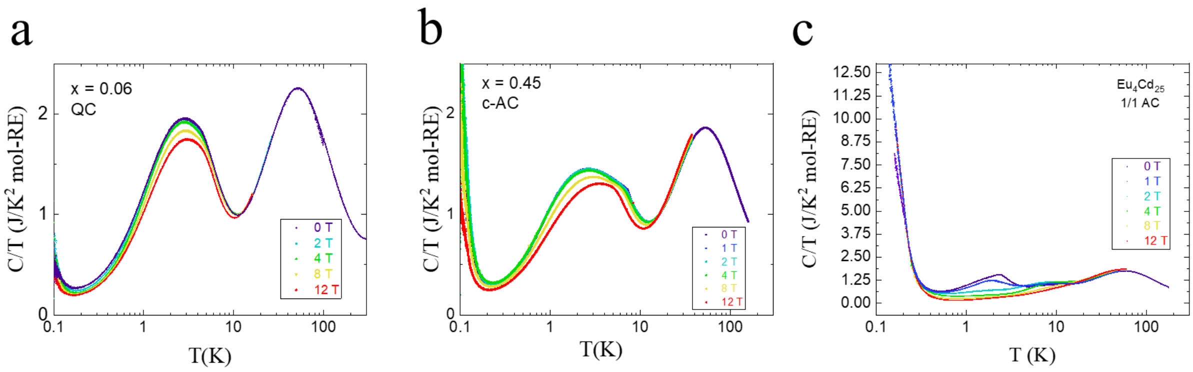

Figure S11: Specific heat, plotted as C/T of (a) the Eu-doped QC sample with concentration x = 0.06, (b) the c-type 1/1 AC with concentration x = 0.45 and (c) Eu_4Cd_{25} with the low temperature

Higher Eu content relates to a larger low-temperature nuclear contribution, with the largest found in the pure Eu_4Cd_{25} 1/1 AC.
